# Plasma Non-Enzymatic Antioxidant Capacity (NEAC) in Relation to Dietary NEAC, Nutrient Antioxidants and Inflammation-Related Biomarkers

**DOI:** 10.3390/antiox9040301

**Published:** 2020-04-05

**Authors:** Cayetano Javier Carrión-García, Eduardo Jesús Guerra-Hernández, Belén García-Villanova, Mauro Serafini, María-José Sánchez, Pilar Amiano, Esther Molina-Montes

**Affiliations:** 1 Department of Nutrition and Bromatology, Faculty of Pharmacy, University of Granada, Campus Universitario de Cartuja S/N, 18071 Granada, Spain; chimo@correo.ugr.es (C.J.C.-G.); belenv@ugr.es (B.G.-V.); 2 Nutrition and Food Science Doctorate Program (RD 99/2011), University of Granada, 18012 Granada, Spain; 3 Functional Food and Metabolic Prevention Lab, Faculty of BioSciences and Technology for Food, Agriculture and Environment, University of Teramo, 64100 Teramo, Italy; mserafini@unite.it; 4 Andalusian School of Public Health, Instituto de Investigación Biosanitaria ibs.GRANADA, Univeristy Hospital of Granada/University of Granada, 18011 Granada, Spain; mariajose.sanchez.easp@juntadeandalucia.es (M.-J.S.); memolina@ugr.es (E.M.-M.); 5 CIBER Epidemiología y Salud Pública, CIBERESP (Centro de Investigación Biomédica), 28029 Madrid, Spain; epicss-san@euskadi.eus; 6 Public Health Division of Gipuzkoa, Biodonostia Health Research Institute, 20014 Donostia-San Sebastian, Spain; 7 Genetic and Molecular Epidemiology Group, Spanish National Cancer Research Centre (CNIO), Madrid, and CIBER Oncology, 28029 Madrid, Spain

**Keywords:** total antioxidant capacity, dietary antioxidants, antioxidant status, oxidative stress, inflammatory markers

## Abstract

(1) Background: Little is known about the interlinkages between dietary and plasma non-enzymatic antioxidant capacity (D-NEAC and P-NEAC, respectively) and the body’s antioxidant and inflammation response. Our aim was to explore these associations in 210 participants from two Spanish European Prospective Investigation into Cancer and Nutrition (EPIC) centers. (2) Methods: D-NEAC was estimated using published NEAC values in food. P-NEAC and total polyphenols (TP) were quantified by FRAP (ferric-reducing antioxidant power), TRAP (total radical-trapping antioxidant parameter), TEAC-ABTS (trolox equivalent antioxidant capacity-Azino Bis Thiazoline Sulfonic), ORAC (oxygen radical absorbance capacity) and Folin–Ciocalteu assays. Nutrient antioxidants (carotenes, α-tocopherol, ascorbic acid, retinol, uric acid, Q9 and Q10 coenzymes) and inflammation markers (IL-6, IL-8, CRP, TNF-α, PAI-I, resistin and adiponectin) were also analyzed. Spearman correlation and linear regression analyses were performed in association analyses. Analyses were stratified by covariates and groups were defined using cluster analysis. (3) Results: P-FRAP was correlated with D-NEAC, and significantly associated with P-NEAC in multivariate adjusted models. P-FRAP levels were also significantly associated with plasma antioxidants (log2 scale: TP β = 0.26; ascorbic acid β = 0.03; retinol β = 0.08; α-tocopherol β = 0.05; carotenes β = 0.02; Q10 β = 0.06; uric acid β = 0.25), though not with inflammation-related biomarkers. Different profiles of individuals with varying levels of P-NEAC and biomarkers were found. (4) Conclusions: P-NEAC levels were to some extent associated with D-NEAC and plasma antioxidants, yet not associated with inflammation response.

## 1. Introduction

An increased production of free radicals and reactive oxygen species (ROS) arise from a variety of sources (endogenous metabolic reactions and exogenous factors, e.g., pollution, smoke or UV irradiation), but living organisms have developed protection mechanisms against oxidative stress (OS) [1,2]. The main mechanism comprises the endogenous enzymatic antioxidant system, but dietary non-enzymatic antioxidants are essential to counteract this process as well. Fruits and vegetables are the main sources of dietary antioxidants including vitamin C, vitamin E and carotenoids [1]. These compounds protect cells from free radical-induced oxidative damage [2,3,4], thereby contributing to reducing the risk of several non-communicable chronic diseases and aging.

Non-enzymatic antioxidant capacity (NEAC) accounts for the cumulative antioxidant capacity of all the antioxidants contained in foods or body fluids, reflecting antioxidant activity and synergistic interactions between these compounds [5]. NEAC is therefore regarded as a global measure of non-enzymatic antioxidant efficiency [6,7]. The main NEAC assays are oxygen radical absorbance capacity (ORAC), total radical-trapping antioxidant parameter (TRAP), trolox equivalent antioxidant capacity-Azino Bis Thiazoline Sulfonic (TEAC-ABTS) and ferric-reducing antioxidant power (FRAP) [5].

Whether plasma NEAC (P-NEAC) or dietary NEAC (D-NEAC) is associated with antioxidant levels in the blood—and in turn with inflammatory markers—is still unknown. This association might depend on how dietary antioxidants interact with each other, and on how they are absorbed and utilized in the body. The gut microbiome is known to modulate the metabolism of nutrient antioxidants, but whether NEAC is affected by certain intestinal bacteria also remains obscure [8]. Little is therefore known about the potential benefits to humans of NEAC. Several studies have analyzed correlation strengths and effect sizes for the association between P-NEAC and inflammation markers [9,10,11]. These studies included less than 100 individuals to assess these associations, with one exception [10]. Thus, these studies may have lacked statistical power to show such associations. Nevertheless, some studies have suggested moderate correlations between C-reactive protein (CRP) and ORAC [10], or CRP and IL-6 with FRAP [9], but not with other inflammation markers. Positive associations have been reported between plasma ORAC and total phenolics (TP), though not with other antioxidants (e.g., α-tocopherol and β-cryptoxanthin) [12]. Other studies also did not observe any associations between P-NEAC and nutrient antioxidants [3,13]. As for D-NEAC, its relationship with P-NEAC has been shown in only a few studies [3,4,14,15,16]. While some associations with biomarkers have been established between D-NEAC and inflammation markers (for example, FRAP with adiponectin [17,18], TEAC with CRP [19], and FRAP, TEAC, ORAC and TRAP with CRP [20]), most studies reported non-significant associations (for example, neither FRAP [17], TEAC [21], nor TRAP [18] were associated with CRP levels). Controversial findings were also reported regarding D-NEAC and nutrient antioxidant marker associations [12,21,22].

Our aim was to explore the association between NEAC and selected nutrient antioxidants and inflammation-related biomarkers in healthy males and females of two EPIC-Spain centers, so as to assess the dietary and plasmatic NEAC´s potential to modulate antioxidant levels in the body, along with the associated inflammation response. The study of such relations is essential to validate the use of dietary and plasma NEAC in aetiological studies.

## 2. Materials and Methods

### 2.1. Study Design

Cross-sectional study.

### 2.2. Study Population 

The study population comprised 210 participants of two Spanish centers of the European Prospective Investigation into Cancer and Nutrition (EPIC) study, EPIC-Granada and EPIC-Gipuzkoa, recruited during 1992–1996 for the EPIC-Spain study. All were healthy (mostly blood donors and volunteers), middle-aged subjects who agreed to participate in the study. The EPIC-Spain study comprising EPIC-Granada, EPIC-Gipuzkoa and another three centers in Spain, was approved by the Research Ethics Committee of the Bellvitge Hospital from Barcelona, where the EPIC-Spain coordination center is located. More details are provided elsewhere [23].

The EPIC study´s major aim is to prospectively investigate diet and cancer associations, which implies a long follow-up and the need for reliable exposure assessments. For the purpose of the current study, involving a validation study of NEAC for its use in future studies, we selected 15,268 subjects from EPIC-Granada and EPIC-Gipuzkoa with blood samples and complete dietary data after applying several exclusion criteria (Figure S1). In brief, we discarded subjects with extreme values of energy intake, self-reported diseases at recruitment and cardiovascular disease risk factors, use of supplements, users of drugs known to promote or alleviate oxidative stress (e.g., aspirin and nonsteroidal anti-inflammatory drugs, among others) [24], and participants who were not at fasting status at blood extraction (defined as more than 6 h fasting). A total of 3732 participants remained, and 210 individuals were selected at random from this pool by stratified sampling by center. To ensure a study sample exposed to a wide range of dietary antioxidants, not only did we consider as strata for sampling the two Spanish centers (of geographical extremes, with varied dietary habits, from north to south) but also quintiles of adherence to the relative Mediterranean diet score, ranging from 0 to 18 points [25]. Thus, 20 individuals were sampled from each quintile in every center (105 samples by center). This study subsample had a sex-distribution close to the original study sample (e.g., 84% and 56% of women in EPIC-Granada and EPIC-Gipuzkoa, respectively). 

### 2.3. Dietary Assessment and D-NEAC Estimation

Dietary data was gathered by means of a validated diet history questionnaire (DHQ) [26]. Briefly, participants were asked about their dietary intake during a typical week over the previous year. Information on food frequency and portion sizes, but also occasional intakes, seasonal differences and variations between working days and weekends was collected. More than 600 food items were introduced in the EPIC nutrient database ENDB [27].

As described elsewhere [4], for the quantification of D-NEAC the United States Department of Agriculture (USDA) database for ORAC and for TP [28], and data of FRAP, TEAC-ABTS and TRAP analyzed in Italian food [29,30] were used. Since coffee is known to have a very high NEAC value due to its high content of TP, but also a poor bioavailability due to the high concentration of Maillard products that inhibit the absorption of polyphenol metabolites [31], we estimated total D-NEAC with and without the contribution of coffee’s NEAC. For ORAC, due to lack of information on ORAC contained in coffee, we previously quantified ORAC in coffee brews [4].

Other variables were collected at recruitment using validated and standardized questionnaires or protocols including lifetime history of smoking consumption, physical activity, and height, weight and waist circumference. More information is provided elsewhere [23]. In relation to physical activity, levels of occupational and recreational physical activity were assigned to metabolic equivalent (METs) per hour and week, and categorized together into inactive, moderately inactive, moderately active and active, based on cut-points determined in the EPIC physical activity validation study [32]. In addition, overall and abdominal obesity were categorized according to the WHO (normal: <25 kg/m^2^; overweight: 25–30 kg/m^2^; obese: ≥30 kg/m^2^) and ATP III criteria (normal: <102 cm in men and <88 cm in women; obese: ≥102 cm in men and ≥88 cm in women), respectively.

### 2.4. Blood Samples and P-NEAC Measurements

Plasma samples were stored in 0.5 mL aliquots in liquid nitrogen (−196 °C) since recruitment. P-NEAC in the form of FRAP, ORAC and TEAC-ABTS was measured with different assays as previously described [4]. Conventional methods, in essence, were applied to measure these NEAC assays [33,34,35]. TRAP was measured as described elsewhere [36]. ORAC without proteins was also measured [37] and Folin–Ciocalteu reagent (FCR) was used to measure TP [38]. A FLUOstar Omega multimode microplate reader (BMG Labtech) was used for the analyses and analytical characteristics [4].

### 2.5. Ascorbic Acid, Dehydroascorbic Acid and Total Vitamin C Determination

Plasma (50 µL) was acidified with an equal volume of 10% (*w*/*v*) meta-phosphoric acid containing 10 mmol/L of disodium-EDTA following centrifugation at 4 °C for 10 min at 11,200× *g*. The amount of 75 µL of supernatant was stored in dark vials at −80 °C until analysis [39]. The quantification was performed by ultra-high-performance liquid chromatography and mass spectrometer Acquity UHPLC BEH system with Waters Xevo TQ-S tandem mass spectrometer (UHPLC-MS/MS) (Waters Co., Milford, MA, USA) and the Acquity UPLC HSS T3 column (2.1 × 100 mm, 1.8 µm). The mobile phase consisted of water 0.1% formic acid and methanol 0.1% formic acid. Sample standards were used. The intra-assay coefficients of variation (CV) were 4.72% for ascorbic acid and 7.36% for dehydroascorbic acid. Inter-assay CVs were 8.65% (range: 4.77–133 µmol/L) for ascorbic acid and 9.01% (range: 0–163 µmol/L) for dehydroascorbic acid. 

### 2.6. Fat-Soluble Antioxidant Compounds Determination

Plasma concentrations of retinol, α-tocopherol, total carotenes, CoQ9 and CoQ10 were likewise determined by UHPLC-MS/MS Acquity and the Acquity UPLC BEH C18 1.7 µm, 2.1 × 100 mm. Briefly, 50 µL of plasma was extracted with 150 µL of 2-propanol containing 0.0625% of BHT following centrifugation at 4 °C for 10 min at 11,200× *g* and 150 µL of supernatant was evaporated with nitrogen and stored in dark vials at −80 °C until analysis [40]. The sample was reconstituted with 150 µL 2-propanol. The mobile phase consisted of 100% methanol 0.1% (*v*/*v*) formic acid. As above, standards were also used. In particular, for total carotenes the standard used was β-carotene. We considered total carotenes because β-carotene was inseparably quantified along with other carotenes in these analyses. Intra-assay CVs were all below 5%. The inter-assay coefficients of variation (CV) were 6.77% (range: 1.34–6.28 µmol/L) for retinol, 8.25% (range: 9.04–128 µmol/L) for α-tocopherol, 9.87% (range: 0.81–87.4 µmol/L) for total carotenes, 9.29% (range: 0.003–0.53 µmol/L) for CoQ9 and 5.11% (range: 0.34–5.31 µmol/L) for CoQ10. 

### 2.7. Inflammation Biomarker Measurements

All biomarkers were analyzed using Luminex 200^TM^ System (Luminex Corporation, Austin, TX, USA). In particular, adiponectin, plasminogen activator inhibitor-1 (PAI-1), and resistin were measured with the Millipore’s MILLIPLEX MAP Human Adipokine Magnetic Bead Panel 1 kit. Interleukin 6 and 8 (IL-6, IL-8), and TNF-α were measured using the Millipore’s MILLIPLEX MAP Human Adipokine Magnetic Bead Panel 2 kit. The intra-assay CVs were all below 10%. Inter-assay CVs were below 15% for adiponectin (range: 0.03–49.9 µg/mL), PAI-I (range: 1.13–78.8 ng/mL), resistin (range: 4.97–83.4 ng/mL), IL-6 (range: 0.01–25.4 pg/mL) and IL-8 (range: 0.24–85.6 pg/mL), and below 20% (range: 0.09–4.00 pg/mL) for TNF-α.

CRP was measured using the MULTIGENT CRP Vario assay (CRPVa) developed and validated for use on the Architect c16000 System (Abbott) for the quantitative immunoturbidimetric determination of this biomarker. Plasma samples were diluted in saline solution to 1:2 dilution factor and antigen-antibody reaction (i.e., agglutination, was detected as an absorbance change (572 nm)). Inter-assay CVs were between 2% (0.45 mg/L values) and 0.26% (45 mg/L values).

### 2.8. Uric Acid Measurements

The clinical chemistry analyzer Mindray BS-200 was used to assess uric acid with a direct colorimetric procedure [41]. Moreover, we removed the contribution of uric acid from P-NEAC levels by subtracting the double value or uric acid from FRAP (2 is the stoichiometric factor of uric acid in the FRAP assay [33]). The inter-assay coefficient of variation (CV) for uric acid was 12.4%.

### 2.9. Statistical Analysis

The data distribution of the plasma biomarkers was tested with kurtosis, skewness and the Shapiro–Wilk test. Since departure from normality was evidenced, log-transformed biomarker values were used in the data analyses. The detection limit divided by two was considered to replace values below the limit of detection if less than 10% of values were below this limit; otherwise, these values were removed.

Spearman correlation (rho) was performed between NEAC and every biomarker. Correlation heatmaps were derived from the correlation matrix.

Multivariate linear regression models were used to analyze the association between P-NEAC and the selected biomarkers, whereby we used their log2 transformation to consider 2-fold increases of the biomarkers in the models. We previously checked that the residuals were normally distributed. Two regression models were performed for each NEAC assay, considered as the dependent variables. In model 1, we controlled for age, sex and center. In model 2 we controlled additionally for lifestyle factors known to affect the antioxidant potential of the diet, namely body mass index (BMI), physical activity and smoking. Other variables, such as season at recruitment, had a negligible impact on the results (estimates changed less than 10% comparing models with and without this variable), and were therefore not considered for additional adjustments. The same regression models were applied to evaluate the association between D-NEAC with the biomarkers. To account for the influence of dietary energy intake, separate models with energy adjustment were considered. To detect the presence of non-linear associations we applied fractional polynomials (mfp package in *R*). Linear associations where thereby verified (fractional power = 1; data not shown).

Hierarchical clustering was applied considering similarity measures (e.g., distance) between each pair of biomarkers (package heatmap in *R*). In particular, we created an unsupervised hierarchy of clusters between the NEAC values and the different biomarkers upon their similarity given by a Manhattan distance matrix [42]. This distance matrix formed the similarity measures used by the hierarchical clustering algorithm. The two most “similar” clusters were joined at each iteration step by the algorithm. Then, the nearest pairs of biomarkers (maximum similarity) were merged into clusters. To calculate the similarity between two clusters, we used Ward´s method. Within the retrieved clusters, we analyzed characteristics by covariates: center, sex, age, body fatness, physical activity and smoking status.

We also evaluated differences between strata of these and other covariates [e.g., waist circumference (abdominal non-obese vs. obese), season at recruitment (spring/summer vs. autumn/winter), and adherence to the Mediterranean Diet (low/medium points vs. high) [25]] in stratified and interaction analyses, whereby an interaction term “biomarker*covariate” was introduced in the regression models. Models with and without the interaction term were compared with the likelihood ratio test (LRT). We adjusted for center in random-effects models since interaction by this covariate was evidenced (package lm4 in *R*).

In sensitivity analyses, we removed influential points (outliers) from the data. These points were identified in multivariate models based on Cook´s distances [43]. We also considered FRAP without the contribution of uric acid to asses uric-acid independent associations, and ORAC without proteins.

All analyses were performed using *R* 3.5.1. software version (R Core Team 2018, Austria, Vienna. http://www.r-project.org/). Two-side tests of significance were considered and *p*-values < 0.05 in hypothesis testing were considered statistically significant.

## 3. Results

Baseline characteristics of the study sample are shown in Table 1. The majority of the participants were women, who were younger, more frequently non-smokers and physically inactive than men (*p*-value < 0.001). In contrast, men had a higher BMI and educational attainment (*p*-value < 0.05) than women. There were also significant differences by sex with regard to energy intake (*p*-value < 0.001) and intake of most antioxidant nutrients and D-NEAC. By center (Table S1), there was a higher proportion of women in the EPIC-Granada center (*p*-value <0.001), and a higher rate of non-formal education, of people who had never smoked and of physically inactive individuals (*p*-value < 0.001). Moreover, there was a significantly higher energy intake in EPIC-Gipuzkoa than in EPIC-Granada (*p*-value < 0.001), possibly driven by the higher proportion of men in EPIC-Gipuzkoa. As a consequence, significantly higher intakes of antioxidants and D-NEAC (*p*-value < 0.001) were observed in this center. Significant differences by center in mean intakes of nutrient antioxidants expressed as nutrient densities (per 1000 Kcal) were kept for most nutrients (Table S1).

Table 2 shows the median and interquartile range of the biomarkers in the study population. Statistically significant differences (*p*-value < 0.001) in median levels of P-NEAC by sex were observed for FRAP with and without uric acid, for TRAP and for ORAC without proteins. We also observed significant sex differences for total carotenes (*p*-value < 0.001), adiponectin (*p*-value = 0.014), PAI-I (*p*-value = 0.008), and resistin (*p*-value = 0.024). There were also statistically significant differences by center (Table S2) regarding some biomarkers (retinol, ascorbic acid, CRP, adiponectin, PAI-I, resistin and IL-8) and P-NEAC including uric acid (*p*-value < 0.001).

**Figure 1:**
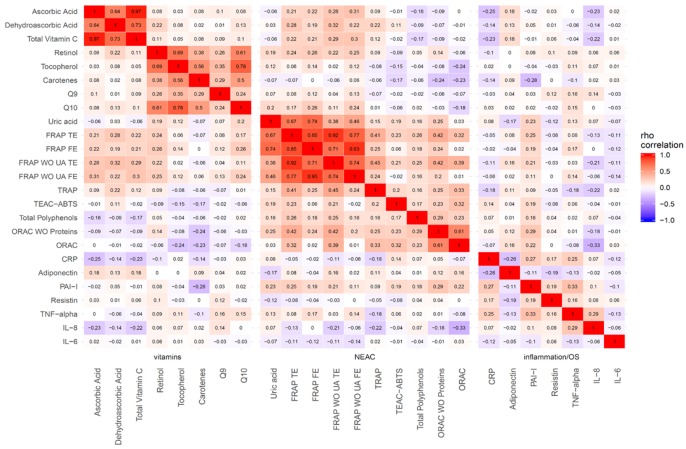
Correlation matrix between biomarkers depicted as a heatmap. (Heat map represents the color-coded correlation factors between all biomarkers including levels of P-NEAC, nutrient antioxidants and biomarkers of inflammation in the EPIC Granada-Gipuzkoa study. The color value of the cells is proportional to the strength of the associations, ranging from red (positive correlations) to blue (negative correlations). The strength of the correlation is indicated in the color scale (at the right of the panel). Pair-wise spearman correlation coefficients (rho) are shown in every cell. Abbreviations: WO = without; UA = uric acid; OS = oxidative stress.

The heatmap of the correlation coefficients between every pair of plasmatic biomarkers is shown in Figure 1. There was a strong correlation (rho > 0.6) between plasma FRAP and uric acid, and milder correlations between FRAP and the other P-NEAC assays (rho > 0.3). Moderately weak correlations were also observed between FRAP and the antioxidants (rho~0.2 to 0.3) and TRAP (rho~0.2 to 0.4), whereas other P-NEAC assays did not seem to correlate with antioxidants despite an overall positive trend between them. Weaker correlations were encountered between P-NEAC levels and adiponectin and non-existent correlations with the other inflammation biomarkers. Importantly, antioxidants were strongly correlated with each other, though less consistently with the inflammation biomarkers. However, there seemed to be a negative trend between nutrient antioxidants and CRP or IL-8 levels (rho~−0.1 to −0.2). Further correlations between D-NEAC and these biomarkers are shown in Table S3. There were positive and significant correlations between dietary FRAP and corresponding levels of plasma FRAP (rho > 0.2), and weaker correlations among the other NEAC assays. Highly significant correlations between D-NEAC and the biomarkers were only observed for ascorbic acid (rho > 0.3). Also, D-NEAC was positively correlated with dietary intake of nutrient antioxidants such as vitamin C, retinol, vitamin E and β-Carotene (rho~0.2 to 0.8) (Table S4).

The association between plasma biomarkers and P-NEAC levels is shown in Table 3 for TRAP and in Table 4 for FRAP. A positive association between plasma TRAP and other P-NEAC assays was seen in age, sex and center-adjusted regression models (e.g., β for log2 FRAP = 0.11; *p*-value = 7.88 × 10^−3^), though not with any of the antioxidant nutrients and inflammation biomarkers. Conversely, significant associations were observed not only between plasma FRAP and TRAP (β for log2 TRAP = 0.15; *p*-value = 7.88 × 10^−3^), but also with regard to TP (β for log2 = 0.26; *p*-value = 4.20 × 10^−4^), and doubling levels (log2) of other nutrient biomarkers: ascorbic acid (β = 0.03; *p*-value = 2.38 × 10^−2^), retinol (β = 0.08; *p*-value = 7.18 × 10^−4^), α-tocopherol (β = 0.05; *p*-value = 2.27 × 10^−3^), total carotenes (β = 0.02; *p*-value =3.78 × 10^−2^), Q10 (β = 0.06; *p*-value = 4.13 × 10^−5^), and uric acid (β = 0.25; *p*-value = 2.02 × 10^−29^). However, there were no significant associations between plasma FRAP levels and inflammation-related biomarkers. The percentage of variance explained by any antioxidant nutrient was higher for FRAP (R2~0.3) than TRAP (R2~0.2). For TEAC-ABTS (Table S5) and ORAC (Table S6), positive and significant associations were observed across other NEAC assays and some (in TEAC-ABTS) or all (in ORAC) nutrient antioxidants. Both plasma TEAC-ABTS and ORAC levels were positively associated with PAI-I (*p*-value = 3.96 × 10^−2^ and 2.26 × 10^−2^, respectively), while high plasma ORAC levels were also associated with decreasing IL-8 (*p*-value = 3.34 × 10^−2^) and increasing adiponectin levels (*p*-value = 0.05). These associations remained in the minimally and multivariate adjusted regression models accounting for the influence of lifestyle factors on the associations, except for IL-8 (with ORAC) and PAI-I (with TEAC-ABTS), which lost statistical significance.

Regarding dietary TRAP and FRAP (Tables S7 and S8, respectively), the association between these NEAC assays and the biomarkers failed to reach statistical significance in multivariate adjusted models. Energy intake had a negligible impact on these associations. Similar results were observed for the other D-NEAC assays (data not shown).

**Figure 2:**
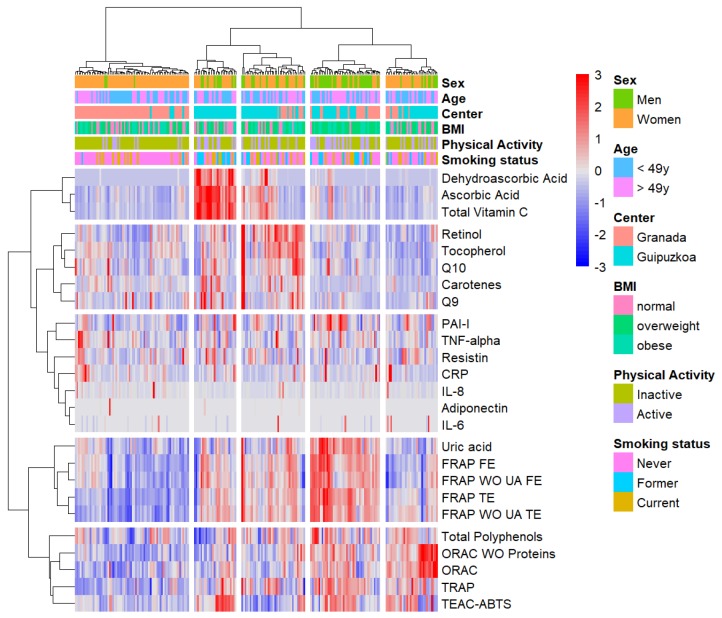
Hierarchical clustering analysis of the analyzed biomarkers across the samples. Unsupervised hierarchical clustering diagram for biomarker levels in 210 samples of the EPIC Granada-Gipuzkoa study. Subjects (samples) were clustered into hierarchical trees based on the levels of the biomarkers, which were clustered by their similarity (Manhattan distances). The clustering separated the biomarkers and subjects into distinct groups. The joined clusters minimized the maximum within-cluster distance. This value is the “height” at which the clusters merged, as indicated in the dendrogram, with height represented on the y-axis. The lower the y-axis value, the lower distance between the clusters and the stronger their relationship. Red indicates high biomarker levels and blue indicates low biomarker levels. Missing data in certain biomarkers appears in white.

Several distinctive clusters were found in the hierarchical clustering (Figure 2). The dendrogram showed that there were five clusters: two antioxidant’s cluster, the inflammation cluster and two NEAC clusters. These clusters correlated with some characteristics of the subjects, giving rise to five clustered patterns. Interestingly, subjects from Gipuzkoa and Granada were fit in separate clusters, while there were two further clusters with a mixed pattern. In the first cluster, there were mainly women of EPIC-Granada exhibiting NEAC and nutrient antioxidants levels below median values. The second cluster featured an opposite pattern and comprised men and women from the EPIC-Gipuzkoa study. The third cluster included subjects from both centers with P-NEAC levels higher than the average but varying levels of vitamin C plasma levels. In the fourth cluster, while nutrient antioxidants levels were typically low, P-NEAC levels were consistently high. In the fifth cluster, a remarkable feature was the relatively higher ORAC and TEAC-ABTS levels compared to low levels of the FRAP and nutrient antioxidants. There was no clear pattern of the subject´s characteristics in these clusters. Indeed, no differences in strata by smoking status (Table S9), BMI (Table S10), and other covariates, including physical activity (data not shown), waist circumference (data not shown), season (data not shown) and adherence to the Mediterranean diet (Table S11) were observed (*p*-value for interaction > 0.05). In general, non-defined patterns were observed in these clusters for the inflammation-related biomarkers. NEAC and biomarker associations were examined in the largest cluster group (cluster-1). Within this cluster, there was no association between the NEAC assays, but a significant association emerged between FRAP and uric acid (β = 0.22), resistin (β = 0.08), CRP (β = 0.03) and adiponectin (β = –0.03) (Table S12).

In sensitivity analyses, we observed to some extent similar results after removing the biomarker´s influential values on the associations (Table S13), and after discounting the contribution of uric acid to the FRAP measure (Table S14).

## 4. Discussion

In this study we sought to examine the association between D-NEAC and P-NEAC with some plasma antioxidants and inflammation markers. Overall, positive though moderate correlations were found among either D-NEAC or P-NEAC assays with nutrient antioxidants, but not with inflammation markers. These associations with nutrient antioxidants hold only for plasma FRAP in analyses adjusted for age, sex, center, physical activity, BMI and smoking status. Clusters of subjects, resulting of combinations of demographic and lifestyle factors according to levels of all biomarkers, suggest that there is variability of P-NEAC in relation to the antioxidant and inflammation status of each individual. 

### 4.1. D-NEAC and P-NEAC Relations

D-NEAC has been associated with health outcomes in several epidemiological studies [44]. However, it has been argued that the in vitro antioxidant capacity may not reflect the real in vivo antioxidant potential of the body [2,5,45]. Studies assessing the relationship between D-NEAC and P-NEAC have reported, at best, moderately-weak correlations for some NEAC assays [4,14,15], while others did not support a correlation [46]. Plasma FRAP, with and without uric acid was positively correlated with dietary FRAP in our study. This result agrees with the study by Pellegrini et al., which reported a moderately-weak correlation between dietary and plasma FRAP in an Italian study population of 285 healthy volunteers [15], as well as with findings of a small study of 50 participants on NEAC diet-plasma relations that we previously conducted [4]. The use of food frequency questionnaires (FFQ) to assess D-NEAC could be a reason for this weak correlation since seasonal variations of antioxidant intakes are not well-captured. However, we have previously shown that both FFQs and 24 h recalls similarly reflect the D-NEAC and P-NEAC relationship [4]. Another possible reason for the seemingly weak correlation between the two is that P-NEAC is affected by many lifestyle factors including dietary habits, the individual´s physiological state, genetic variation and gut microbiome composition and function [47]. All of them have a well-known direct impact on the mechanisms of digestion, absorption and metabolism of dietary antioxidants, resulting in a high inter-individual variability of P-NEAC [45]. For instance, P-NEAC has been found to be higher in smokers compared to non-smokers [5], in men [18] and in overweight/obese individuals [12]. These studies had a small sample size and have not been replicated in other studies, or in our study. Our results may be determined by this P-NEAC variability, as we did not observe an association between dietary and plasma FRAP when accounting for lifestyle factors in multivariate regression models. The somewhat weak correlations of D-NEAC and P-NEAC could also be due to the influence of other antioxidant compounds in body fluids, such as proteins for ORAC and uric acid for FRAP [5]. In our study, all associations were kept when removing their effect on the NEAC assays. The low bioavailability of some antioxidants (e.g., flavonoids) only reach the nanomolar range in plasma [48], could also explain the low correlation strength. Moreover, the majority of these NEAC assays are performed in aqueous solutions, which implies that hydrophobic antioxidants or insoluble antioxidants can be underestimated [6]. 

### 4.2. Dietary/Plasma NEAC Associations with Nutrient/Inflammation Markers

In our study, there was no association between either D-NEAC and P-NEAC or CRP levels. Likewise, no association was found between dietary FRAP and CRP levels among 532 healthy adults participating in the ATTICA study [18], or among 4506 participants from the Rotterdam study [17]. ORAC plasma levels were also not associated with CRP levels in a study carried out among 815 Spanish individuals [10]. The lack of an overall association in our study could be explained by the fact that levels of CRP fell within a low and narrow range. Indeed, our study population comprised healthy subjects only, less likely to overproduce this marker. However, there are also studies reporting an association between D-NEAC and CRP levels among healthy individuals, such as the study by Kobayashi et al. that included 474 Japanese women [20], and the study by Brighenti et al. of 243 Italian non-diabetic subjects [19]. Importantly, this latter study also showed that the association was higher for subjects with hypertension than in normo-tensive individuals, suggesting that the association between D-NEAC and plasma CRP may strengthen under pro-inflammatory conditions. Other previous studies have also reported that a low intake of dietary antioxidants is associated with elevated inflammatory parameters, supporting that inflammation may underpin mechanisms linking antioxidants and OS with disease outcomes. The study by Wang et al., for instance, showed that a high D-NEAC intake was associated with lower plasma CRP levels in 35 postmenopausal and overweight/obese women [12]. High P-NEAC levels (FRAP) were also inversely associated with CRP levels among 80 patients with End-Stage Renal Failure [9]. Thus, if antioxidants foods are related to a low inflammatory profile in low-grade chronic inflammation conditions (e.g., smokers, obese and diseased individuals) and/or in a healthy state keeps being an unresolved issue. Our study did also not support an association between NEAC and the other inflammatory markers. With regard to studies that evaluated TNF-α, IL-6, PAI-I, resistin and adiponectin in relation to D-NEAC or P-NEAC levels [9,11,12,17], only the Rotterdam study found significant inverse associations between dietary FRAP and PAI-I and a positive association between dietary FRAP and adiponectin [17]. 

With regard to nutrient antioxidant biomarkers, high P-NEAC as FRAP was associated with high levels of almost all antioxidants, including TP. While this marker seems to not reflect the amount of phenolic compounds contained in food [45], we observed positive associations between all P-NEAC assays and TP. D-NEAC was also positively associated with dietary intake (all carotenoids and flavonoids) and plasma antioxidant levels (lutein and α-tocopherol) in a study of 60 healthy non-smoking subjects [3]. However, contrary to our study, higher NEAC levels (FRAP and VCEAC) were not significantly related to increasing levels of ascorbic acid or TP in plasma. The study by Wang et al. conducted among postmenopausal women also did not show significant associations between D-NEAC/P-NEAC (ORAC, FRAP and VCEAC) and dietary intake of nutrient antioxidants [12]. Our study is therefore the first unravelling an association between both D-NEAC/P-NEAC and nutrient antioxidant status.

While our results did not support different effect measures between D-NEAC/P-NEAC and the biomarkers by sex, smoking status, obesity or physical activity (except center), there were distinctive cluster groups of individuals. These clusters may reflect different patterns regarding the relationship between P-NEAC and the nutrient and inflammation markers. Our study sample was too small to observe differing association patterns across the subgroups or among individuals more prone to inflammatory states, except within the largest cluster group. In this cluster, featuring non-smoking and inactive women mainly, the trend went in the opposite direction for some inflammatory markers (adiponectin, resistin and CRP). 

One of the main limitations was we could not assess the association between D-NEAC and P-NEAC with OS markers. Antioxidant enzymes playing a key role in the antioxidant defenses of the body were also not considered. However, their impact on D-NEAC uptake is unclear. In fact, several studies did not observe a significant change in their activity according to the intake of dietary antioxidants, foods or supplements [49,50]. The degree of their activity depends on the individual´s genotype [51,52], which may also have affected our results. Non-nutrient antioxidants not accounted for in this study could have also influenced this association by activating pathways connected to the endogenous defense and immune system [53]. We also had no measurements of other inflammatory markers such as leptin, but to the best of our knowledge, this study has considered the largest set of markers. Our results are therefore consistent with the absence of an association between an antioxidant-rich diet and a low inflammatory state. However, we cannot fully rule out this association given the small sample size. Other limitations are related to the study design (causal associations cannot be drawn), residual confounding (the influence of other covariates on the associations cannot be precluded), generalizability of the results (their extrapolation to other populations cannot be established), and a single biomarker assessment (variations of the associations over time cannot be assessed). 

Regarding strengths, we used a DHQ administered by in-person interviews, and were able to minimize measurement error in reporting the intake of anti-oxidant rich foods thanks to the fact that information was collected on seasonal variations in the patterns of dietary intake, added fats, recipes and dishes combining foods [23], among other issues. Measurement errors are also unlikely in our biomarker determinations as all values fell in the expected range (e.g., BMI-adiponectin correlation; rho = –0.63). Since there is a well-known variability of P-NEAC levels [45], we examined how lifestyle and external factors could influence the associations. This is the first study demonstrating that there are, indeed, many different profiles of subjects with varying relations between P-NEAC and the biomarkers. Since coffee’s NEAC can confuse the total D-NEAC estimates [48,54], we considered both total D-NEAC and D-NEAC without the contribution of coffee’s NEAC.

## 5. Conclusions

Findings from this study suggest that D-NEAC is related to P-NEAC to a weak extent. Only P-NEAC, most likely FRAP, was positively associated with nutrient antioxidant levels in plasma, whereas no association was observed with inflammation biomarkers. Thus, plasma FRAP may best reflect the antioxidant potential of the human body but seem to not have anti-inflammatory effects in healthy subjects. Our results also suggest that there may be subgroups of individuals with low diet/plasma NEAC response against inflammation. The latter may comprise high-risk groups with antioxidant depletion who are eligible for dietary/lifestyle interventions. Larger studies are warranted to reexamine the existence of such groups and to validate these findings.

## Figures and Tables

**Table 1:** Baseline characteristics of the study sample (63 males and 147 females) within the EPIC Granada–Gipuzkoa study.

Variables	All (*n* = 210)	Men (*n* = 63)	Women (*n* = 147)	*p*-Value ^2^
	Median [IQR 25–75]	Median [IQR 25–75]	Median [IQR 25–75]	
**Age**	48.8 [41.4;55.2]	51.9 [46.6;57.1]	46.3 [39.9;53.1]	0.001
**BMI (kg/m^2^)**	27.2 [24.8;30.4]	28 [25.9;30.7]	26.4 [24.2;30.2]	0.042
**Cigarettes/day among smokers**	10.0 [5.00;20.0]	17.5 [7.00;20.0]	9.00 [3.00;12.0]	0.103
	***N* (%)**	***N* (%)**	***N* (%)**	
**Abdominal obesity**				0.781
Normal	128 (60.9)	37 (58.7)	91 (61.9)	
Obese	82 (39.1)	26 (41.3)	56 (38.1)	
**Center**				<0.001
Granada	105 (50.0)	17 (27.0)	88 (59.9)	
Gipuzkoa	105 (50.0)	46 (73.0)	59 (40.1)	
**Smoking status**				
Never smoker	130 (61.9)	25 (39.7)	105 (71.4)	<0.001
Former smoker	34 (16.2)	19 (30.2)	15 (10.2)	
Current smoker	45 (21.4)	19 (30.2)	26 (17.7)	
**Physical activity**				
Inactive	78 (37.1)	5 (7.94)	73 (49.7)	<0.001
Moderately inactive	68 (32.4)	19 (30.2)	49 (33.3)	
Moderately active	41 (19.5)	24 (38.7)	17 (11.6)	
Active	23 (11.0)	15 (23.8)	8 (5.44)	
**Education Level**				
None	72 (34.6)	13 (21.0)	59 (40.4)	0.088
Primary school	90 (43.3)	34 (54.8)	56 (38.4)	
Secondary school	16 (7.70)	5 (8.06)	11 (7.53)	
Professional	12 (5.80)	2 (3.23)	10 (6.85)	
University	18 (8.70)	8 (12.9)	10 (6.85)	
**Dietary Characteristics ^1^**	**Median [IQR 25–75]**	**Median [IQR 25–75]**	**Median [IQR 25–75]**	
Energy intake (kcal/day)	1875 [1548;2310]	2432 [2175;2934]	1712 [1421;1999]	<0.001
Fruits (g/day)	254 [139;409]	280 [142;436]	250 [139;395]	0.294
Vegetables (g/day)	206 [123;297]	211 [118;313]	206 [132;285]	0.954
Legumes (g/day)	37.2 [23.3;63.4]	48.0 [29.8;102]	35.0 [22.6;51.2]	0.001
Cereals (g/day)	202 [135;260]	261 [202;326]	173 [127;232]	<0.001
Meat and meat products (g/day)	101 [69.2;148]	140 [102;163]	89.6 [65.3;120]	<0.001
Fish and seafood (g/day)	54.3 [32.3;83.2]	79.7 [49.3;120]	49.3 [27.2;69.9]	<0.001
Milk and dairy products (g/day)	253 [161;376]	246 [150;333]	258 [166;399]	0.232
Red wine (g/day)	0.00 [0.00;40.2]	100 [0.00;192]	0.00 [0.00;0.00]	<0.001
Coffee (g/day)	84.4 [3.36;152]	98.3 [26.8;131]	76.8 [2.93;174]	0.901
Tea (g/day)	0.00 [0.00;0.00]	0.00 [0.00;0.00]	0.00 [0.00;0.00]	0.684
Flavonoids (mg/1000 Kcal/day)	161 [111;227]	170 [115;227]	157 [107;225]	0.385
β-Carotene (µg/1000 Kcal/day)	1092 [719;1574]	922 [565;1436]	1221 [836;1601]	0.005
Retinol (µg/ 1000 Kcal/day)	145 [93.8;204]	117 [88.5;183]	149 [95.8;207]	0.075
α-Tocopherol (mg/1000 Kcal/day)	5.7 [4.5;7.5]	5.2 [4.5;7]	6.1 [7.5;7.7]	0.094
Vitamin C (mg/1000 Kcal/day)	66.6 [44.8;89.5]	56.8 [40.4;71.6]	71.6 [46.3;102]	<0.001
Iron (mg/1000 Kcal/day)	7.2 [6.3;8.3]	7.32 [6.67;8.34]	7.10 [6.06;8.21]	0.153
Alcohol (g/1000 Kcal/day)	0.89 [0.01;5.9]	8.67 [2.88;15.6]	0.11 [0.00;1.99]	<0.001
TRAP (µmol TE/day)	8990 [3764;15,231]	10,830 [5663;1,6160]	8083 [3431;14,836]	0.025
TRAP _without coffee_ (µmol TE/day)	2771 [1876;4631]	4584 [3103;7025]	2285 [1748;3548]	<0.001
FRAP (µmol Fe^2+^/day)	22,388 [11,079;33,821]	26,226 [17,529;36,363]	19,713 [9421;33,458]	0.009
FRAP _without coffee_ (µmol Fe^2+^/day)	8765 [6560;13,636]	14,221 [10,581;17,979]	7720 [5948;11,355]	<0.001
TEAC-ABTS (µmol TE/day)	6855 [3625;10,336]	8304 [5844;11,608]	6130 [3176;10,050]	0.003
TEAC-ABTS _without coffee_ (µmol TE/day)	3083 [2321;4828]	4791 [3417;6665]	2739 [2037;4002]	<0.001
ORAC (µmol TE/day)	31,501 [15,818;48,097]	33,193 [21,398;44,293]	28,174 [14,444;49,880]	0.286
ORAC _without coffee_ (µmol TE/day)	12,042 [8597;16,299]	14,300 [10,447;19,927]	11,338 [8040;15,434]	<0.001
Total Polyphenols _without coffee_ (mgGAE/day)	1519 [1108;2033]	1760 [1386;2511]	1433 [1056;1938]	<0.001

^1^ Dietary data were derived from the diet history questionnaire. Missing data: smoking status (*n* = 1). ^2^ Student’s *t* test and Wilcoxon’s test for continuous variables, where appropriate, and chi-square test for categorical variables. TRAP: total radical-trapping antioxidant parameter; FRAP: ferric-reducing antioxidant power; TEAC-ABTS: trolox equivalent antioxidant capacity—Azino Bis Thiazoline Sulfonic; ORAC: oxygen radical absorbance capacity; TE: Trolox equivalents.

**Table 2:** Plasma biomarker levels in the study sample (63 males and 147 females) within the EPIC Granada–Gipuzkoa study.

Biomarkes	All			Men		Women		
	*N* = 210			*N* = 63		*N* = 147		
	Median	IQR (25–75)	N	Median	IQR (25–75)	Median	IQR (25–75)	*p*-Value ^1^
Ascorbic acid (µmol/L)	27.8	[20.9;46.3]	210	28.2	[23.9;54.0]	27.8	[20.4;42.7]	0.201
Dehydroascorbic acid (µmol/L)	0.00	[0.00;3.76]	210	0.00	[0.00;6.85]	0.00	[0.00;0.00]	0.007
Total vitamin C (µmol/L)	29.3	[21.4;52.5]	210	31.6	[24.3;64.3]	28.9	[20.4;44.5]	0.113
Retinol (µmol/L)	2.22	[1.85;2.73]	210	2.31	[1.94;2.81]	2.16	[1.79;2.66]	0.066
Tocopherol (µmol/L)	28.4	[21.6;37.3]	210	27.3	[20.7;37.0]	29.4	[21.7;37.5]	0.410
Carotenes (µmol/L)	3.47	[2.34;6.73]	210	2.79	[1.80;4.27]	3.92	[2.62;7.65]	<0.001
Q9 (µmol/L)	0.05	[0.03;0.08]	210	0.05	[0.03;0.09]	0.05	[0.03;0.07]	0.727
Q10 (µmol/L)	1.16	[0.96;1.50]	210	1.17	[1.00;1.59]	1.15	[0.95;1.50]	0.662
Uric acid (mg/dl)	3.73	[3.05;4.48]	210	4.71	[3.96;5.41]	3.45	[2.92;4.03]	<0.001
FRAP (µmol TE/L)	457	[403;519]	210	528	[471;560]	428	[393;476]	<0.001
FRAP (µmol Fe^2+^/L)	881	[808;982]	210	996	[910;1079]	853	[785;924]	<0.001
FRAP without uric ccid (µmol TE/L)	314	[267;355]	210	345	[312;387]	296	[257;341]	<0.001
FRAP without uric acid (µmol Fe^2+^/L)	634	[574;699]	210	692	[630;754]	616	[556;678]	<0.001
TRAP (µmol TE/L)	976	[884;1073]	210	1034	[907;1116]	949	[867;1047]	0.001
TEAC-ABTS (µmol TE/L)	3041	[2599;3677]	210	3115	[2508;3823]	3008	[2647;3384]	0.642
Total polyphenols (mg GAE/L)	1207	[1128;1276]	210	1206	[1126;1270]	1207	[1132;1277]	0.850
ORAC without proteins (µmol TE/L)	1160	[946;1399]	210	1308	[1098;1555]	1124	[908;1358]	<0.001
ORAC (µmol TE/L)	14,706	[12,739;17,005]	210	15,138	[13,185;17,622]	14,547	[12,617;16,648]	0.173
CRP (mg/L)	1.26	[0.76;2.38]	207	1.18	[0.76;2.15]	1.32	[0.76;2.50]	0.299
Adiponectin (µg/mL)	0.10	[0.07;0.15]	207	0,08	[0.06;0.11]	0.10	[0.07;0.17]	0.014
PAI-I (ng/mL)	20.0	[14.6;27.0]	210	22.9	[17.9;29.8]	19.4	[14.3;25.2]	0.008
Resistin (ng/mL)	14.2	[11.5;18.2]	210	13.3	[10.5;16.1]	14.4	[12.0;18.8]	0.024
TNF-α (pg/mL)	0.75	[0.58;1.00]	162	0.77	[0.62;1.06]	0.74	[0.51;0.98]	0.323
IL-8 (pg/mL)	1.08	[0.68;1.70]	146	1.09	[0.68;1.68]	1.06	[0.69;1.71]	0.850
IL-6 (pg/mL)	0.69	[0.69;0.69]	210	0.69	[0.69;0.69]	0.69	[0.69;0.69]	0.776

IQR = P25–P75 ^1^ Wilcoxon’s test for continuous variables. CRP: C-reactive protein; PAI-I: Plasminogen activator inhibitor-1; TNF-α: Tumor necrosis factor.

**Table 3:** Association between P-NEAC as TRAP and the nutrient/inflammation biomarkers in the EPIC Granada–Gipuzkoa cohort sub-sample (*n* = 210).

Biomarkers	Model 1					Model 2				
	β Coefficient	CI 95%		*p*-Value	*R^2^*	β Coefficient	CI 95%		*p*-Value	*R^2^*
Ascorbic acid (µmol/L)	−0.018	−0.038	0.002	8.47 × 10^−2^	0.199	−0.020	−0.040	0.001	6.16 × 10^−2^	0.219
Dehydroascorbic acid (µmol/L)	0.000	−0.001	0.001	9.33 × 10^−1^	0.187	0.000	−0.001	0.001	9.71 × 10^−1^	0.205
Total vitamin C (µmol/L)	−0.011	−0.029	0.007	2.22 × 10^−1^	0.193	−0.013	−0.031	0.006	1.70 × 10^−1^	0.212
Retinol (µmol/L)	−0.010	−0.051	0.031	6.30 × 10^−1^	0.188	−0.009	−0.051	0.033	6.68 × 10^−1^	0.206
α-Tocopherol (µmol/L)	−0.013	−0.041	0.015	3.68 × 10^−1^	0.190	−0.015	−0.043	0.014	3.17 × 10^−1^	0.209
Carotenes (µmol/L)	−0.002	−0.016	0.012	8.23 × 10^−1^	0.187	−0.003	−0.017	0.012	6.90 × 10^−1^	0.206
Q9 (µmol/L)	−0.010	−0.024	0.004	1.80 × 10^−1^	0.194	−0.011	−0.026	0.003	1.27 × 10^−1^	0.214
Q10 (µmol/L)	−0.001	−0.027	0.025	9.55 × 10^−1^	0.187	−0.002	−0.029	0.025	9.1 × 10^−1^	0.205
Uric acid (mg/dl)	0.042	0.000	0.084	5.33 × 10^−2^	0.202	0.046	0.000	0.091	5.03 × 10^−2^	0.220
FRAP (µmol TE/L)	0.164	0.091	0.238	**1.86 × 10^−5^**	0.257	0.166	0.090	0.243	**3.25 × 10^−5^**	0.271
FRAP (µmol Fe^2+^/L)	0.110	0.030	0.190	**7.88 × 10^−3^**	0.215	0.102	0.019	0.186	**1.73 × 10^−2^**	0.227
FRAP without uric ccid (µmol TE/L)	0.143	0.079	0.207	**1.84 × 10^−5^**	0.257	0.139	0.074	0.205	**4.54 × 10^−5^**	0.269
FRAP without uric acid (µmol Fe^2+^/L)	0.083	0.010	0.155	**2.59 × 10^−2^**	0.206	0.072	−0.002	0.147	5.85 × 10^−2^	0.219
TEAC-ABTS (µmol TE/L)	0.046	0.003	0.088	**3.80 × 10^−2^**	0.204	0.051	0.007	0.094	**2.49 × 10^−2^**	0.225
Total polyphenols (mg GAE/L)	0.144	0.018	0.269	**2.58 × 10^−2^**	0.206	0.146	0.012	0.280	**3.45 × 10^−2^**	0.223
ORAC without proteins (µmol TE/L)	0.033	−0.008	0.075	1.18 × 10^−1^	0.197	0.039	−0.004	0.081	7.78 × 10^−2^	0.217
ORAC (µmol TE/L)	0.077	0.021	0.133	**7.45 × 10^−3^**	0.215	0.079	0.022	0.135	**7.29 × 10^−3^**	0.233
CRP (mg/L)	−0.004	−0.016	0.009	5.78 × 10^−1^	0.187	−0.004	−0.018	0.010	5.43 × 10^−1^	0.205
Adiponectin (µg/mL)	0.006	−0.008	0.020	3.95 × 10^−1^	0.195	0.007	−0.008	0.021	3.69 × 10^−1^	0.216
PAI-I (ng/mL)	0.007	−0.017	0.031	5.61 × 10^−1^	0.188	0.008	−0.016	0.033	5.14 × 10^−1^	0.207
Resistin (ng/mL)	−0.025	−0.056	0.006	1.19 × 10^−1^	0.197	−0.025	−0.057	0.007	1.24 × 10^−1^	0.214
TNF-α (pg/mL)	−0.008	−0.035	0.019	5.55 × 10^−1^	0.202	−0.008	−0.036	0.019	5.55 × 10^−1^	0.219
IL-8 (pg/mL)	−0.006	−0.023	0.010	4.50 × 10^−1^	0.254	−0.006	−0.022	0.011	5.26 × 10^−1^	0.278
IL-6 (pg/mL)	−0.001	−0.017	0.015	8.93 × 10^−1^	0.187	−0.002	−0.018	0.015	8.19 × 10^−1^	0.205

Plasma biomarkers were log2 transformed. Model 1: Multiple linear regression adjusted for age (continuous, years), sex (male, females) and center (Granada, Gipuzkoa). Model 2: Multiple linear regression adjusted age (continuous, years), sex (male, females) and center (Granada, Gipuzkoa), BMI (continuous, kg/m2), physical activity (inactive, moderately inactive and active, active) and smoking status (never, former, current smoker). Statistically significant associations are shown in bold.

**Table 4:** Association between P-NEAC as FRAP and the nutrient/inflammation biomarkers in the EPIC Granada–Gipuzkoa cohort sub-sample (*n* = 210).

Biomarkers	Model 1					Model 2				
	β Coefficient	CI 95%		*p*-Value	*R^2^*	β Coeff	CI 95%		*p*-Value	*R^2^*
**Ascorbic Acid (µmol/L)**	**0.027**	0.004	0.051	**2.38 × 10^−2^**	0.247	0.027	0.003	0.050	**2.81 × 10^−2^**	0.292
Dehydroascorbic acid (µmol/L)	0.001	0.000	0.001	1.73 × 10^−1^	0.235	0.001	0.000	0.001	1.64 × 10^−1^	0.281
Total vitamin C (µmol/L)	0.017	−0.004	0.038	1.07 × 10^−1^	0.238	0.017	−0.004	0.038	1.21 × 10^−1^	0.283
Retinol (µmol/L)	0.081	0.035	0.127	**7.18 × 10^−4^**	0.270	0.080	0.033	0.127	**9.92 × 10^−4^**	0.313
α-Tocopherol (µmol/L)	0.050	0.018	0.082	**2.27 × 10^−3^**	0.263	0.050	0.018	0.082	**2.77 × 10^−3^**	0.306
Carotenes (µmol/L)	0.017	0.001	0.033	**3.78 × 10^−2^**	0.244	0.017	0.001	0.034	**3.76 × 10^−2^**	0.290
Q9 (µmol/L)	0.013	−0.003	0.030	1.12 × 10^−1^	0.238	0.011	−0.005	0.028	1.90 × 10^−1^	0.280
Q10 (µmol/L)	0.062	0.033	0.091	**4.13 × 10^−5^**	0.289	0.062	0.032	0.092	**6.43 × 10^−5^**	0.331
Uric acid (mg/dl)	0.245	0.209	0.282	**2.02 × 10^−29^**	0.585	0.252	0.213	0.291	**1.06 × 10^−27^**	0.603
TRAP (µmol TE/L)	0.149	0.040	0.257	**7.88 × 10^−3^**	0.254	0.133	0.024	0.242	**1.73 × 10^−2^**	0.295
TEAC-ABTS (µmol TE/L)	−0.015	−0.066	0.035	5.49 × 10^−1^	0.230	−0.018	−0.068	0.033	4.90 × 10^−1^	0.276
Total polyphenols (mg GAE/L)	0.262	0.119	0.406	**4.20 × 10^−4^**	0.274	0.246	0.095	0.397	**1.65 × 10^−3^**	0.310
ORAC without proteins (µmol TE/L)	0.033	−0.016	0.082	1.85 × 10^−1^	0.235	0.031	−0.019	0.079	2.24 × 10^−1^	0.280
ORAC (µmol TE/L)	−0.037	−0.103	0.029	2.72 × 10^−1^	0.233	−0.043	−0.109	0.023	2.02 × 10^−1^	0.280
CRP (mg/L)	0.002	−0.013	0.016	8.37 × 10^−1^	0.229	−0.004	−0.019	0.012	6.44 × 10^−1^	0.277
Adiponectin (µg/mL)	−0.005	−0.021	0.011	5.29 × 10^−1^	0.231	−0.002	−0.018	0.014	8.11 × 10^−1^	0.276
PAI-I (ng/mL)	0.019	−0.009	0.046	1.88 × 10^−1^	0.235	0.010	−0.018	0.039	4.68 × 10^−1^	0.276
Resistin (ng/mL)	0.011	−0.026	0.047	5.73 × 10^−1^	0.229	0.007	−0.030	0.043	7.24 × 10^−1^	0.275
TNF-α (pg/mL)	0.022	−0.009	0.053	1.68 × 10^−1^	0.207	0.016	−0.015	0.047	3.14 × 10^−1^	0.252
IL-8 (pg/mL)	−0.003	−0.023	0.016	7.40 × 10^−1^	0.201	−0.008	−0.028	0.012	4.40 × 10^−1^	0.232
IL-6 (pg/mL)	−0.016	−0.035	0.002	8.73 × 10^−2^	0.239	−0.016	−0.035	0.002	8.97 × 10^−2^	0.285

Plasma biomarkers were log2 transformed. Model 1: Multiple linear regression adjusted for age (continuous, years), sex (male, females) and center (Granada, Gipuzkoa). Model 2: Multiple linear regression adjusted age (continuous, years), sex (male, females) and center (Granada, Gipuzkoa), BMI (continuous, kg/m2), physical activity (inactive, moderately inactive and active, active) and smoking status (never, former, current smoker). Statistically significant associations are shown in bold.

## Supplementary Materials

The following are available online at https://www.mdpi.com/2076-3921/9/4/301/s1, **Figure S1:** Flow chart illustrating the process applied to select 210 study participants. **Table S1:** Baseline characteristics of the study sample (63 males and 147 females) within the EPIC Granada–Gipuzkoa study by center. **Table S2:** Plasma biomarker levels in the study sample (63 males and 147 females) within the EPIC Granada–Gipuzkoa study by center. **Table S3:** Correlation coefficients between D-NEAC and every biomarker in the EPIC Granada–Gipuzkoa sub-sample (*n* = 210). **Table S4:** Correlation coefficients between dietary antioxidant vitamins and D-NEAC, P-NEAC and plasma antioxidant vitamins in the EPIC Granada–Gipuzkoa sub-sample (*n* = 210). **Table S5:** Association between P-NEAC as TEAC-ABTS and biomarkers in the EPIC Granada–Gipuzkoa sub-sample (*n* = 210). **Table S6:** Association between P-NEAC as ORAC and biomarkers in the EPIC Granada–Gipuzkoa sub-sample (*n* = 210). **Table S7:** Association between D-NEAC as TRAP without coffee and biomarkers in the EPIC Granada–Gipuzkoa sub-sample (*n* = 210) in multivariate models. **Table S8:** Association between D-NEAC as FRAP without coffee and biomarkers in the EPIC Granada–Gipuzkoa sub-sample (*n* = 210) in multivariate models. **Table S9:** Association between P-NEAC as FRAP and biomarkers in the EPIC Granada–Gipuzkoa sub-sample (*n* = 210) by smoking status. **Table S10:** Association between P-NEAC as FRAP and biomarkers in the EPIC Granada–Gipuzkoa sub-sample (*n* = 210) by body mass index (BMI). **Table S11:** Association between P-NEAC as FRAP and biomarkers in the EPIC Granada–Gipuzkoa sub-sample (*n* = 210) by body levels of adherence to the Mediterranean diet. **Table S12:** Association between P-NEAC as FRAP and biomarkers in cluster-1, comprising mostly women from EPIC-Granada, who were non-smokers and inactive, *n* = 70. Median FRAP levels = 395.0 µmol TE/L. **Table S13:** Sensitivity analyses comprising the removal of influential points based on Cook´s distances in multivariate models. **Table S14:** Association between P-NEAC as FRAP without uric acid and biomarkers in the EPIC Granada–Gipuzkoa cohort sub-sample (*n* = 210).
