# Supplementary material for: Plasma Non-Enzymatic Antioxidant Capacity (NEAC) in Relation to Dietary NEAC, Nutrient Antioxidants and Inflammation-Related Biomarkers
Source: Antioxidants (Basel). 2020 Apr 5;9(4):301. doi: 10.3390/antiox9040301 (PMC7222170; doi:10.3390/antiox9040301)
Supplement: Supplementary file 1 [file antioxidants-09-00301-s001.pdf]

Supplementary material:

Figure S1: Flow chart illustrating the process applied to select 210 study participants.

Table S1: Baseline characteristics of the study sample (63 males and 147 females) within the EPIC Granada-Gipuzkoa study by centre.

Table S2: Plasma biomarker levels in the study sample (63 males and 147 females) within the EPIC Granada-Gipuzkoa study by centre.

Table S3: Correlation coefficients between D-NEAC and every biomarker in the EPIC Granada-Gipuzkoa sub-sample (n=210).

Table S4: Correlation coefficients between dietary antioxidant vitamins and D-NEAC, P-NEAC and plasma antioxidant vitamins in the EPIC Granada-Gipuzkoa sub-sample (n=210).

Table S5: Association between P-NEAC as TEAC-ABTS and biomarkers in the EPIC Granada-Gipuzkoa sub-sample (n=210).

Table S6: Association between P-NEAC as ORAC and biomarkers in the EPIC Granada-Gipuzkoa sub-sample (n=210).

Table S7: Association between D-NEAC as TRAP without coffee and biomarkers in the EPIC Granada-Gipuzkoa sub-sample (n=210) in multivariate models.

Table S8: Association between D-NEAC as FRAP without coffee and biomarkers in the EPIC Granada-Gipuzkoa sub-sample (n=210) in multivariate models.

Table S9: Association between P-NEAC as FRAP and biomarkers in the EPIC Granada-Gipuzkoa sub-sample (n=210) by smoking status.

Table S10: Association between P-NEAC as FRAP and biomarkers in the EPIC Granada-Gipuzkoa sub-sample (n=210) by body mass index (BMI).

Table S11: Association between P-NEAC as FRAP and biomarkers in the EPIC Granada-Gipuzkoa sub-sample (n=210) by levels of adherence to the Mediterranean Diet.

Table S12: Association between P-NEAC as FRAP and biomarkers in cluster-1, comprising mostly women from EPIC-Granada, who were non-smokers and inactive, n=70. Median FRAP levels = 395.0 µmol TE/L.

Table S13: Sensitivity analyses comprising the removal of influential points based on Cook´s distances in multivariate models.

Table S14: Association between P-NEAC as FRAP without uric acid and biomarkers in the EPIC Granada-Gipuzkoa cohort sub-sample (n=210).

Figure S1: Flow chart illustrating the process applied to select 210 study participants.

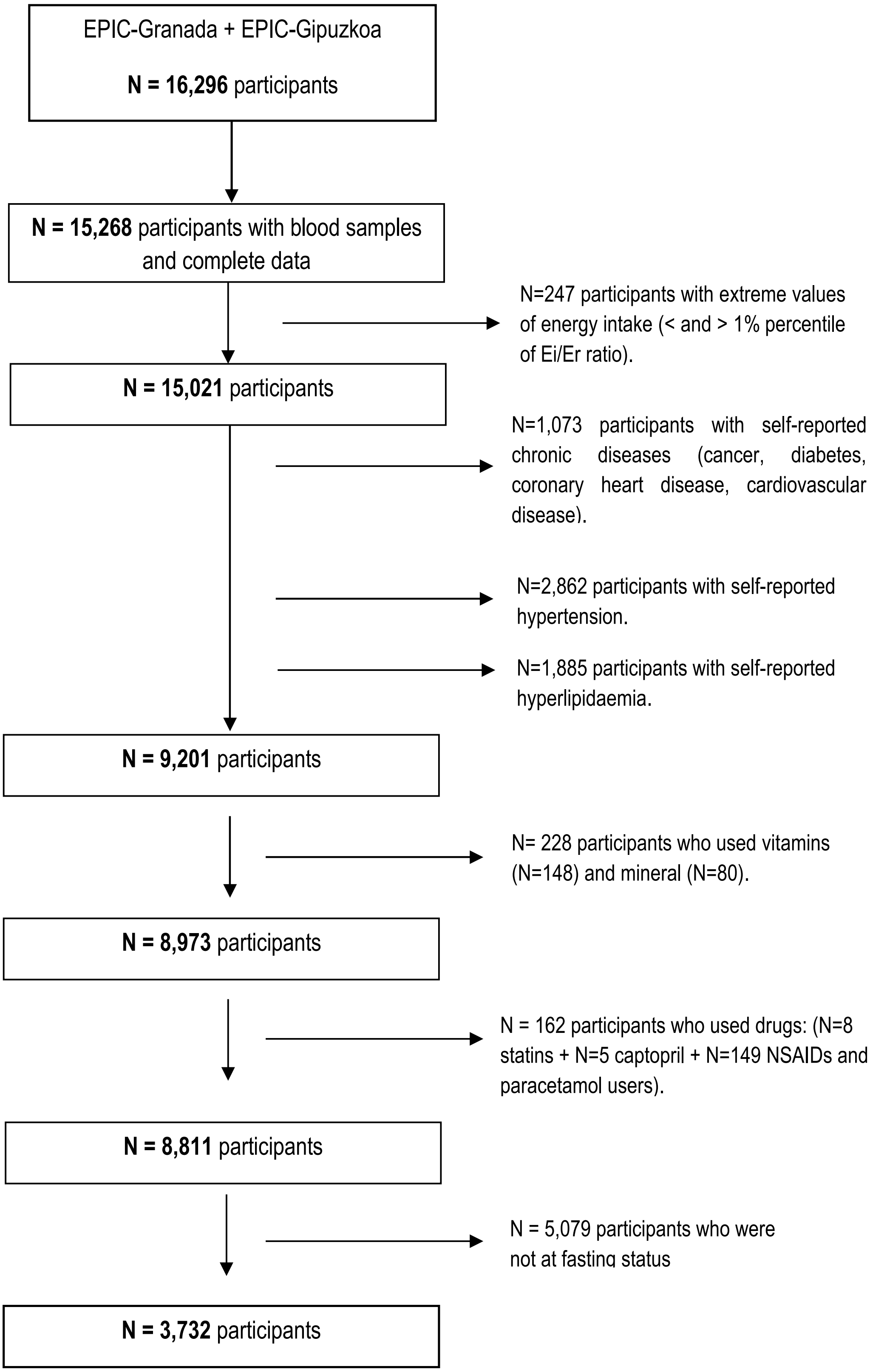

Table S1: Baseline characteristics of the study sample (63 males and 147 females) within the EPIC Granada-Gipuzkoa study by centre.

**Variables**	**All (n=210) Median [IQR 25-75]**	**Granada (n=105) Median [IQR 25-75]**	**Gipuzkoa (n=105) Median [IQR 25-75]**	***p-value*[2]**
Age	48.8 [41.4;55.2]	50.0 [40.1;58.7]	48.0 [41.9;52.8]	0.417
BMI (kg/m^2)	27.2 [24.8;30.4]	27.7 [25.1;31.4]	26.6 [24.3;29.2]	0.041
Cigarettes/day among smokers	10.0 [5.00;20.0]	9.50 [5.50;19.7]	15.0 [4.50;20.0]	0.214
	**N (%)**	**N (%)**	**N (%)**	
Abdominal obesity				0.066
Normal	128 (69.9)	57 (54.3)	71 (67.6)	
Obese	82 (31.1)	48 (45.7)	34 (32.4)	
Sex				
Men	63 (30.0)	17 (16.2)	46 (43.8)	<0.001
Women	147 (70.0)	88 (83.8)	59 (56.2)	
Smoking status				
Never smoker	130 (61.9)	77 (73.3)	53 (50.5)	0.003
Former smoker	34 (16.2)	11 (10.5)	23 (21.9)	
Current smoker	45 (21.4)	17 (16.2)	28 (26.7)	
Physical activity				
Inactive	78 (37.1)	53 (50.5)	25 (23.8)	<0.001
Moderately inactive	68 (32.4)	34 (32.4)	34 (32.4)	
Moderately active	41 (19.5)	10 (9.52)	31 (29.5)	
Active	23 (11.0)	8 (7.62)	15 (14.3)	
Education Level				
None	72 (34.6)	48 (46.6)	24 (22.9)	0.001
Primary school	90 (43.3)	32 (31.1)	58 (55.2)	
Secondary school	16 (7.70)	5 (4.85)	11 (10.5)	
Professional	12 (5.80)	7 (6.80)	5 (4.76)	
University	18 (8.70)	11 (10.7)	7 (6.67)	
**Dietary Characteristics**[1]	**Median [IQR 25-75]**	**Median [IQR 25-75]**	**Median [IQR 25-75]**	
Energy intake (kcal/day)	1875 [1548;2310]	1633 [1406;1956]	2178 [1834;2591]	<0.001
Fruits (g/day)	254 [139;405]	224 [133;342]	289 [137;447]	0.155
Vegetables (g/day)	206 [123;297]	197 [119;271]	216 [139;327]	0.144
Legumes (g/day)	37.2 [23.3;63.4]	35.0 [24.3;48.6]	45.1 [23.3;85.7]	0.001
Cereals (g/day)	202 [135;260]	188 [126;242.0]	213 [155;271]	0.009
Meat and meat products (g/day)	101 [69.2;148]	85.4 [60.7;112]	124 [83.3;161]	<0.001
Fish and seafood (g/day)	54.3 [32.3;83.2]	49.3 [28.3;74.1]	62.7 [43.6;109]	0.001
Milk and dairy products (g/day)	253 [161;376]	260 [168;371]	250 [157;396]	0.673
Red wine (g/day)	0.00 [0.00;40.2]	0.00 [0.00;0.00]	0.00 [0.00;139]	<0.001
Coffee (g/day)	84.4 [3.36;152]	27.0 [1.29;100]	125 [57.1;196]	<0.001
Tea (g/day)	0.00 [0.00;0.00]	0.00 [0.00;0.00]	0.00 [0.00;0.00]	0.964
Flavonoids (mg/1000 Kcal/day)	161 [111;227]	157 [106;238]	164 [112;223]	0.943
β-Carotene (µg/1000 Kcal/day)	1092 [719;1574]	1069 [781;1503]	1158 [671;1671]	0.853
Retinol (µg/1000 Kcal/day)	145 [93.8;204]	144 [94.0;182]	151 [93.7;240]	0.141
α-Tocopherol (mg/1000 Kcal/day)	5.7 [4.5;7.5]	5.48 [4.41;6.99]	6.09 [4.51;7.93]	0.075
Vitamin C (mg/1000 Kcal/day)	66.6 [44.8;89.5]	73.5 [48.8;102]	59.6 [41.3;80.4]	0.008
Iron (mg/1000 Kcal/day)	7.2 [6.3;8.3]	6.8 [6;8]	7.6 [6.8;8.5]	0.002
Alcohol (g/1000 Kcal/day)	0.89 [0.01;5.9]	0.06 [0.00;1.02]	4.24 [0.71;10.1]	<0.001
TRAP (µmol TE/day)	8990 [3764;15231]	5102 [2606;10789]	12201 [7797;17526]	<0.001
TRAP $_{\text{without coffee}}$ (µmol TE/day)	2771 [1876;4631]	2285 [1752;3524]	3597 [2262;5198]	<0.001
FRAP (µmol Fe^{2+}/day)	22388 [11079;33821]	13559 [8412;25517]	29230 [20427;38316]	<0.001
FRAP $_{\text{without coffee}}$ (µmol Fe^{2+}/day)	8765 [6560;13636]	7995 [6039;11404]	11411 [7617;15826]	<0.001
TEAC-ABTS (µmol TE/day)	6855 [3625;10336]	4465 [2826;7849]	9271 [6346;11529]	<0.001
TEAC-ABTS $_{\text{without coffee}}$ (µmol TE/day)	3083 [2321;4828]	2816 [2077;3979]	4042 [2610;5657]	<0.001
ORAC (µmol TE/day)	31501 [15818;48097]	18769 [11338;38710]	41473 [27696;58649]	<0.001
ORAC $_{\text{without coffee}}$ (µmol TE/day)	12042 [8597;16299]	10804 [7837;14300]	13765 [10049;18615]	<0.001
Total Polyphenols $_{\text{without coffee}}$ (mg GAE/day)	1519 [1108;2033]	1442 [1053;1910]	1630 [1277;2168]	0.005

[1] Dietary data were derived from the diet history questionnaire. [2] Student's *t* test and Wilcoxon's test for continuous variables, where appropriate, and chi-square test for categorical variables. Missing data: smoking status (n=1).

Table S2: Plasma biomarker levels in the study sample (63 males and 147 females) within the EPIC Granada-Gipuzkoa study by centre.

Plasma Biomarkers and P-NEAC	All N=210 Median	IQR (25-75)	N	Granada N=105 Median	IQR (25-75)	Gipuzkoa N=105 Median	IQR (25-75)	p-value
Ascorbic Acid (µmol/L)	27.8	[20.9;46.3]	210	24.0	[17.9;30.4]	42.1	[27.2;68.5]	<0.001
Dehydroascorbic Acid (µmol/L)	0.00	[0.00;3.76]	210	0.00	[0.00;0.00]	3.49	[0.00;20.9]	<0.001
Total Vitamin C (µmol/L)	29.3	[21.4;52.5]	210	24.3	[17.9;30.4]	47.4	[28.7;93.2]	<0.001
Retinol (µmol/L)	2.22	[1.85;2.73]	210	2.10	[1.72;2.45]	2.38	[1.98;2.87]	<0.001
Tocopherol (µmol/L)	28.4	[21.6;37.3]	210	29.6	[22.9;36.2]	27.2	[20.4;39.2]	0.692
Carotenes (µmol/L)	3.47	[2.34;6.73]	210	3.80	[2.52;6.74]	3.18	[2.14;6.68]	0.244
Q9 (µmol/L)	0.05	[0.03;0.08]	210	0.04	[0.03;0.07]	0.05	[0.03;0.10]	0.212
Q10 (µmol/L)	1.16	[0.96;1.50]	210	1.15	[0.95;1.41]	1.17	[1.00;1.69]	0.272
Uric Acid (mg/dl)	3.73	[3.05;4.48]	210	3.63	[3.08;4.37]	3.78	[2.97;4.84]	0.764
FRAP (µmol TE/L)	457	[403;519]	210	416	[383;478]	484	[444;543]	<0.001
FRAP (µmol Fe^{2+}/L)	881	[808;982]	210	860	[782;954]	917	[837;1023]	0.001
FRAP without Uric Acid (µmol TE/L)	314	[267;355]	210	272	[249;318]	338	[309;378]	<0.001
FRAP without Uric Acid (µmol Fe^{2+}/L)	634	[574;699]	210	604	[545;680]	662	[600;730]	<0.001
TRAP (µmol TE/L)	976	[884;1073]	210	901	[852;1008]	1044	[938;1121]	<0.001
TEAC-ABTS (µmol TE/L)	3041	[2599;3677]	210	2947	[2737;3230]	3152	[2403;4227]	0.150
Total Polyphenols (mg GAE/L)	1207	[1128;1276]	210	1206	[1127;1261]	1209	[1130;1287]	0.533
ORAC without proteins (µmol TE/L)	1160	[946;1399]	210	1077	[907;1284]	1280	[1094;1550]	<0.001
ORAC (µmol TE/L)	14706	[12739;17005]	210	14055	[11883;15971]	15463	[13995;17966]	<0.001
CRP (mg/L)	1.26	[0.76;2.38]	207	1.79	[1.04;3.13]	1.02	[0.52;1.94]	<0.001
Adiponectin (µg/ml)	0.10	[0.07;0.15]	207	0.08	[0.05;0.14]	0.11	[0.08;0.15]	0.002
PAI-I (ng/ml)	20.0	[14.6;27.0]	210	19.4	[14.3;24.3]	22.3	[15.1;30.1]	0.016
Resistin (ng/ml)	14.2	[11.5;18.2]	210	13.5	[10.8;16.7]	15.1	[12.0;19.2]	0.012
TNF-α (pg/ml)	0.75	[0.58;1.00]	162	0.80	[0.62;1.07]	0.70	[0.50;0.95]	0.058
IL-8 (pg/ml)	1.08	[0.68;1.70]	146	1.27	[0.90;2.05]	0.87	[0.60;1.31]	0.001
IL-6 (pg/ml)	0.69	[0.69;0.69]	210	0.69	[0.69;0.69]	0.69	[0.69;0.69]	0.788

IQR= P25-P75

Wilcoxon's test for continuous variables.

Table S3: Correlation coefficients between D-NEAC and every biomarker in the EPIC Granada-Gipuzkoa sub-sample (n=210).

Biomarkers	FRAP	FRAP w/o coffee	TRAP	TRAP w/o coffee	TEAC-ABTS	TEAC-ABTS w/o coffee	ORAC	ORAC w/o coffe	TP w/o coffee
**Plasma biomarkers and P-NEAC**									
Ascorbic Acid (μmol/L)	0.30**	0.16*	0.29**	0.14*	0.29**	0.15*	0.31**	0.21*	0.20*
Dehydroascorbic Acid (μmol/L)	0.30**	0.14*	0.29**	0.15*	0.29**	0.13	0.31**	0.19*	0.17*
Total Vitamin C (μmol/L)	0.32**	0.16*	0.31**	0.14*	0.32**	0.15*	0.34**	0.22*	0.21*
Retinol (μmol/L)	0.05	0.02	0.05	0.02	0.05	0.02	0.04	-0.02	-0.01
α-Tocopherol (μmol/L)	0.00	-0.09	0.00	-0.09	-0.01	-0.07	0.03	-0.03	0.00
Carotenes (μmol/L)	-0.11	-0.07	-0.12	-0.09	-0.12	-0.07	-0.03	0.08	0.12
Q9 (μmol/L)	-0.02	0.01	-0.04	-0.02	-0.02	0.04	-0.02	0.01	0.03
Q0 (μmol/L)	0.02	-0.04	0.02	-0.04	0.01	-0.03	0.03	-0.05	-0.03
Uric Acid (mg/dl)	0.15*	0.18*	0.14*	0.16*	0.16*	0.17*	0.10	0.09	0.08
FRAP (μmol TE/L)	0.32**	0.29**	0.31**	0.27**	0.32**	0.26**	0.26**	0.20*	0.17*
FRAP (μmol Fe^{2+}/L)	0.18*	0.25**	0.17*	0.23**	0.19*	0.23**	0.11	0.15*	0.14*
FRAP without Uric Acid (μmol TE/L)	0.35**	0.29**	0.33**	0.27**	0.34**	0.26**	0.29**	0.22*	0.19*
FRAP without Uric Acid (μmol Fe^{2+}/L)	0.18*	0.24**	0.16*	0.22*	0.18*	0.22*	0.10	0.13	0.13
TRAP (μmol TE/L)	0.12	0.18*	0.11	0.18*	0.10	0.14*	0.09	0.17*	0.14*
TEAC-ABTS (μmol TE/L)	0.07	0.07	0.06	0.06	0.08	0.09	0.03	0.05	0.03
Total Polyphenols (mg GAE/L)	-0.16*	-0.13	-0.14*	-0.12	-0.17*	-0.17*	-0.14*	-0.08	-0.08
ORAC without proteins (μmol TE/L)	0.22*	0.13	0.23*	0.15*	0.22*	0.12	0.17*	0.05	0.00
ORAC (μmol TE/L)	0.17*	0.15*	0.18*	0.16*	0.17*	0.14*	0.15*	0.10	0.06
CRP (mg/L)	-0.09	-0.12	-0.08	-0.09	-0.07	-0.08	-0.10	-0.10	-0.08
Adiponectin (μg/ml)	0.19*	0.00	0.20*	0.01	0.18*	-0.01	0.21*	0.04	0.03
PAI-I (ng/ml)	0.19*	0.10	0.19*	0.12	0.20*	0.11	0.14*	0.01	0.00
Resistin (ng/ml)	-0.05	-0.12	-0.03	-0.06	-0.04	-0.11	-0.03	-0.06	-0.08
TNF-α (pg/ml)	0.02	0.05	0.00	0.02	0.02	0.06	-0.04	-0.06	-0.04
IL-8 (pg/ml)	-0.15	-0.14	-0.15	-0.14	-0.14	-0.12	-0.15	-0.18*	-0.14
IL-6 (pg/ml)	-0.05	0.05	-0.06	0.07	-0.05	0.03	0.00	0.16*	0.15*
**D-NEAC**									
FRAP (μmol Fe^{2+}/day)	-	0.42**	0.99**	0.41**	0.99**	0.42**	0.94**	0.31**	0.28**
FRAP without coffee (μmol Fe^{2+}/day)	0.42**	-	0.35**	0.97**	0.47**	0.98**	0.24**	0.64**	0.64**
TRAP (μmol TE/day)	0.99**	0.35**	-	0.37**	0.98**	0.36**	0.94**	0.25**	0.21*
TRAP without coffee (μmol TE/day)	0.41**	0.97**	0.37**	-	0.47**	0.96**	0.23**	0.59**	0.59**
TEAC-ABTS (μmol TE/day)	0.99**	0.47**	0.98**	0.47**	-	0.49**	0.92**	0.34**	0.31**
TEAC-ABTS without coffee (μmol TE/day)	0.42**	0.98**	0.36**	0.96**	0.49**	-	0.25**	0.62**	0.63**
ORAC (μmol TE/day)	0.94**	0.24**	0.94**	0.23**	0.92**	0.25**	-	0.37**	0.34**
ORAC without coffee (μmol TE/day)	0.31**	0.64**	0.25**	0.59**	0.34**	0.62**	0.37**	-	0.98**
Total Polyphenols without coffee (mg GAE/day)	0.28**	0.64**	0.21*	0.59**	0.31**	0.63**	0.34**	0.98**	-

Nonparametric Spearman's correlation. * Positive statistically significant correlation coefficients (p<0.05); ** Positive statistically significant correlation coefficients (p<0.001).

Table S4: Correlation coefficients between dietary antioxidant vitamins, D-NEAC, P-NEAC and plasma antioxidant vitamins in the EPIC Granada-Gipuzkoa sub-sample (n=210).

Biomarkers	Vitamin C [1]	Retinol [1]	Vitamin E [1]	β-Carotene [1]
**Plasma biomarkers and P-NEAC**				
Ascorbic Acid (µmol/L)	0.16*	0.14*	0.26**	0.25**
Dehydroascorbic Acid (µmol/L)	0.11	0.18*	0.26**	0.18*
Total Vitamin C (µmol/L)	0.18*	0.16*	0.26**	0.24**
Retinol (µmol/L)	0.04	0.03	0.08	0.02
α-Tocopherol (µmol/L)	0.11	-0.15*	-0.04	-0.05
Carotenes (µmol/L)	0.23**	-0.11	0.05	0.15*
FRAP (µmol TE/L)	0.06	0.17*	0.28**	0.00
FRAP (µmol Fe^{2+}/L)	0.06	0.12	0.20*	-0.03
FRAP without Uric Acid (µmol TE/L)	0.09	0.18*	0.29**	0.05
FRAP without Uric Acid (µmol Fe^{2+}/L)	0.08	0.15*	0.17*	0.00
TRAP (µmol TE/L)	0.02	0.10	0.27**	0.14
TEAC-ABTS (µmol TE/L)	-0.09	-0.02	0.02	-0.04
Total Polyphenols (mg GAE/L)	-0.12	-0.07	-0.12	-0.20*
ORAC without proteins (µmol TE/L)	-0.13	0.12	0.09	-0.08
ORAC (µmol TE/L)	-0.02	0.07	0.19*	0.05
**D-NEAC**				
FRAP (µmol Fe^{2+}/day)	0.19*	0.20*	0.33**	0.27**
FRAP without coffee (µmol Fe^{2+}/day)	0.52**	0.09	0.46**	0.45**
TRAP (µmol TE/day)	0.12	0.20*	0.29**	0.23*
TRAP without coffee (µmol TE/day)	0.45**	0.07	0.43**	0.42**
TEAC-ABTS (µmol TE/day)	0.21*	0.20*	0.34**	0.29**
TEAC-ABTS without coffee (µmol TE/day)	0.52**	0.10	0.44**	0.45**
ORAC (µmol TE/day)	0.24**	0.17*	0.33**	0.27**
ORAC without coffee (µmol TE/day	0.75**	0.01	0.54**	0.54**
Total Polyphenols without coffee (mgGAE/day)	0.85**	-0.03	0.52**	0.56**
**Dietary vitamins**				
Vitamin C (mg/day)	-	-0.09	0.40**	0.54**
Retinol (µg/day)	-0.09	-	0.33**	0.13
Vitamin E (mg/day)	0.40**	0.33**	-	0.50**
β-Carotene (µg/day)	0.54**	0.13	0.50**	-

Nonparametric Spearman's correlation.

* Positive statistically significant correlation coefficients ($p<0.05$); ** Positive statistically significant correlation coefficients ($p<0.001$).

[1]Dietary vitamins.

Table S5: Association between P-NEAC as TEAC-ABTS and biomarkers in the EPIC Granada-Gipuzkoa sub-sample (n=210).

Biomarkers	Model 1					Model 2				
	β coeff	CI 95%		p-value	R^2	β coeff	CI 95%		p-value	R^2
Ascorbic Acid (µmol/L)	-0.015	-0.060	0.030	5.09E-01	0.020	-0.020	-0.065	0.025	3.92E-01	0.060
Dehydroascorbic Acid (µmol/L)	-0.000	-0.002	0.001	8.05E-01	0.018	-0.000	-0.002	0.001	6.81E-01	0.058
Total Vitamin C (µmol/L)	-0.009	-0.048	0.031	6.63E-01	0.018	-0.014	-0.054	0.027	5.07E-01	0.059
Retinol (µmol/L)	0.118	0.030	0.206	**9.25E-03**	0.049	0.132	0.042	0.222	**4.64E-03**	0.094
α-Tocopherol (µmol/L)	0.081	0.020	0.141	**9.61E-03**	0.049	0.084	0.022	0.145	**8.30E-03**	0.090
Carotenes (µmol/L)	0.034	0.003	0.064	**3.08E-02**	0.040	0.033	0.002	0.065	**3.68E-02**	0.077
Q9 (µmol/L)	-0.017	-0.048	0.015	2.97E-01	0.023	-0.020	-0.051	0.012	2.18E-01	0.064
Q10 (µmol/L)	-0.035	-0.092	0.022	2.31E-01	0.024	-0.037	-0.095	0.022	2.24E-01	0.064
Uric Acid (mg/dl)	0.118	0.027	0.210	**1.23E-02**	0.047	0.113	0.014	0.211	**2.58E-02**	0.080
FRAP (µmol TE/L)	0.202	0.036	0.368	**1.82E-02**	0.044	0.208	0.036	0.380	**1.88E-02**	0.083
FRAP (µmol Fe^{2+}/L)	-0.055	-0.234	0.124	5.49E-01	0.019	-0.065	-0.249	0.119	4.90E-01	0.059
FRAP without Uric Acid (µmol TE/L)	0.141	-0.005	0.286	5.96E-02	0.034	0.153	0.006	0.300	**4.26E-02**	0.076
FRAP without Uric Acid (µmol Fe^{2+}/L)	-0.165	-0.325	-0.006	**4.34E-02**	0.037	-0.161	-0.323	0.000	5.25E-02	0.075
TRAP (µmol TE/L)	0.220	0.013	0.426	**3.80E-02**	0.038	0.239	0.032	0.447	**2.49E-02**	0.081
Total Polyphenols (mg GAE/L)	0.309	0.033	0.585	**2.92E-02**	0.040	0.401	0.110	0.691	**7.44E-03**	0.090
ORAC without proteins (µmol TE/L)	0.100	0.009	0.191	**3.22E-02**	0.039	0.099	0.006	0.192	**3.76E-02**	0.077
ORAC (µmol TE/L)	0.233	0.113	0.354	**1.91E-04**	0.082	0.242	0.120	0.363	**1.28E-04**	0.124
CRP (mg/L)	0.034	0.007	0.062	**1.54E-02**	0.044	0.030	0.000	0.060	5.47E-02	0.080
Adiponectin (µg/ml)	0.011	-0.020	0.042	5.03E-01	0.021	0.011	-0.021	0.042	4.97E-01	0.064
PAI-I (ng/ml)	0.054	0.003	0.106	**3.96E-02**	0.038	0.052	-0.001	0.105	5.70E-02	0.074
Resistin (ng/ml)	-0.051	-0.119	0.018	1.50E-01	0.027	-0.054	-0.123	0.015	1.28E-01	0.068
TNF-α (pg/ml)	0.006	-0.054	0.066	8.43E-01	0.025	-0.006	-0.066	0.055	8.50E-01	0.091
IL-8 (pg/ml)	0.005	-0.033	0.043	7.93E-01	0.022	0.003	-0.036	0.042	8.82E-01	0.052
IL-6 (pg/ml)	-0.002	-0.037	0.034	9.31E-01	0.017	0.005	-0.031	0.041	7.83E-01	0.057

Plasma biomarkers were log2 transformed.

Model 1: Multiple linear regression adjusted for age (continuous, years), sex (male, females) and centre (Granada, Gipuzkoa).

Model 2: Multiple linear regression adjusted age (continuous, years), sex (male, females) and centre (Granada, Gipuzkoa), BMI (continuous, kg/m^2), physical activity (inactive, moderately inactive and active, active) and smoking status (never, former, current smoker).

Positive association are shown in bold.

Table S6: Association between P-NEAC as ORAC and biomarkers in the EPIC Granada-Gipuzkoa sub-sample (n=210).

Biomarkers	Model 1					Model 2				
	**β coeff**	**CI 95%**		**p-value**	**R^2**	**β coeff**	**CI 95%**		**p-value**	**R^2**
Ascorbic Acid (μmol/L)	0.060	0.027	0.093	**4.20E-04**	0.168	0.058	0.025	0.092	**8.55E-04**	0.186
Dehydroascorbic Acid (μmol/L)	0.002	0.001	0.003	**4.19E-04**	0.168	0.002	0.001	0.002	**4.97E-04**	0.191
Total Vitamin C (μmol/L)	0.063	0.034	0.092	**2.72E-05**	0.188	0.061	0.031	0.091	**7.85E-05**	0.205
Retinol (μmol/L)	0.081	0.014	0.149	**1.92E-02**	0.139	0.088	0.019	0.158	**1.37E-02**	0.165
α-Tocopherol (μmol/L)	0.080	0.034	0.125	**7.39E-04**	0.163	0.083	0.036	0.130	**5.92E-04**	0.189
Carotenes (μmol/L)	0.037	0.015	0.060	**1.55E-03**	0.158	0.041	0.018	0.065	**6.67E-04**	0.188
Q9 (μmol/L)	0.017	-0.041	0.007	1.63E-01	0.124	0.017	-0.041	0.007	1.74E-01	0.147
Q10 (μmol/L)	0.061	0.018	0.104	**5.52E-03**	0.148	0.073	0.029	0.117	**1.47E-03**	0.182
Uric Acid (mg/dl)	0.007	0.064	0.078	8.39E-01	0.116	-0.006	-0.082	0.070	8.76E-01	0.139
FRAP (μmol TE/L)	0.174	0.048	0.301	**7.39E-03**	0.146	0.171	0.039	0.303	**1.17E-02**	0.167
FRAP (μmol Fe^{2+}/L)	-0.076	-0.213	0.060	2.72E-01	0.121	-0.092	-0.233	0.049	2.02E-01	0.146
FRAP without Uric Acid (μmol TE/L)	0.194	0.085	0.302	**5.88E-04**	0.165	0.194	0.083	0.305	**7.15E-04**	0.189
FRAP without Uric Acid (μmol Fe^{2+}/L)	-0.094	-0.216	0.028	1.32E-01	0.125	-0.099	-0.224	0.026	1.21E-01	0.150
TRAP (μmol TE/L)	0.215	0.059	0.371	**7.45E-03**	0.146	0.219	0.061	0.377	**7.29E-03**	0.170
TEAC-ABTS (μmol TE/L)	0.136	0.066	0.206	**1.91E-04**	0.174	0.142	0.071	0.214	**1.28E-04**	0.201
Total Polyphenols (mg GAE/L)	0.320	0.112	0.529	**2.88E-03**	0.153	0.280	0.057	0.504	**1.49E-02**	0.165
CRP (mg/L)	0.005	-0.016	0.027	6.24E-01	0.112	0.003	-0.020	0.027	7.99E-01	0.135
Adiponectin (μg/ml)	0.024	0.000	0.048	**5.01E-02**	0.132	0.028	0.004	0.052	**2.49E-02**	0.162
PAI-I (ng/ml)	0.046	0.007	0.085	**2.26E-02**	0.138	0.044	0.003	0.085	**3.49E-02**	0.159
Resistin (ng/ml)	-0.014	-0.067	0.039	6.06E-01	0.117	-0.014	-0.067	0.040	6.15E-01	0.140
TNF-α (pg/ml)	-0.015	-0.059	0.029	4.96E-01	0.105	-0.017	-0.062	0.029	4.78E-01	0.130
IL-8 (pg/ml)	-0.031	-0.059	-0.003	**3.34E-02**	0.127	-0.028	-0.056	0.001	5.89E-02	0.172
IL-6 (pg/ml)	0.002	-0.025	0.029	8.78E-01	0.116	0.001	-0.027	0.029	9.47E-01	0.139

Plasma biomarkers were log2 transformed.

Model 1: Multiple linear regression adjusted for age (continuous, years), sex (male, females) and centre (Granada, Gipuzkoa).

Model 2: Multiple linear regression adjusted age (continuous, years), sex (male, females) and centre (Granada, Gipuzkoa), BMI (continuous, kg/m^2), physical activity (inactive, moderately inactive and active, active) and smoking status (never, former, current smoker).

Positive association are shown in bold.

Table S7: Association between D-NEAC as TRAP without coffee and biomarkers in the EPIC Granada-Gipuzkoa sub-sample (n=210) in multivariate models.

Biomarkers	Model 1					Model 2					Model 3				
	β coeff	CI 95%		p-value	R^2	β coeff	CI 95%		p-value	R^2	β coeff	CI 95%		p-value	R^2
Ascorbic Acid (µmol/L)	0.025	-0.075	0.125	6.26E-01	0.243	0.015	-0.084	0.115	7.62E-01	0.300	0.005	-0.091	0.100	9.22E-01	0.359
Dehydroascorbic Acid (µmol/L)	-0.001	-0.004	0.002	3.85E-01	0.245	-0.001	-0.004	0.002	4.06E-01	0.302	-0.002	-0.004	0.001	3.15E-01	0.362
Total Vitamin C (µmol/L)	0.014	-0.074	0.102	7.57E-01	0.242	0.007	-0.081	0.095	8.77E-01	0.300	-0.007	-0.092	0.078	8.71E-01	0.359
Retinol (µmol/L)	-0.111	-0.311	0.089	2.78E-01	0.246	-0.098	-0.299	0.104	3.43E-01	0.303	-0.099	-0.292	0.095	3.20E-01	0.362
α-Tocopherol (µmol/L)	-0.073	-0.210	0.065	3.01E-01	0.246	-0.063	-0.201	0.074	3.67E-01	0.303	-0.047	-0.179	0.085	4.87E-01	0.360
Carotenes (µmol/L)	0.010	-0.059	0.078	7.78E-01	0.242	0.007	-0.062	0.076	8.46E-01	0.300	-0.007	-0.074	0.060	8.38E-01	0.359
Q9 (µmol/L)	-0.032	-0.101	0.038	3.75E-01	0.245	-0.030	-0.099	0.039	3.95E-01	0.302	-0.015	-0.082	0.051	6.51E-01	0.360
Q10 (µmol/L)	-0.064	-0.191	0.064	3.28E-01	0.245	-0.039	-0.168	0.091	5.61E-01	0.301	-0.032	-0.156	0.092	6.14E-01	0.360
Uric Acid (mg/dl)	-0.121	-0.329	0.087	2.56E-01	0.247	-0.017	-0.235	0.202	8.82E-01	0.300	-0.017	-0.227	0.193	8.73E-01	0.359
FRAP (µmol TE/L)	0.032	-0.345	0.410	8.67E-01	0.242	0.180	-0.203	0.562	3.58E-01	0.303	0.134	-0.234	0.501	4.76E-01	0.361
FRAP (µmol Fe^{2+}/L)	-0.066	-0.467	0.335	7.48E-01	0.242	0.076	-0.329	0.480	7.14E-01	0.300	-0.003	-0.393	0.387	9.89E-01	0.359
FRAP without Uric Acid (µmol TE/L)	0.100	-0.229	0.428	5.53E-01	0.243	0.170	-0.156	0.495	3.07E-01	0.304	0.118	-0.196	0.431	4.63E-01	0.361
FRAP without Uric Acid (µmol Fe^{2+}/L)	0.006	-0.354	0.367	9.73E-01	0.242	0.079	-0.280	0.438	6.67E-01	0.301	-0.011	-0.358	0.336	9.49E-01	0.359
TRAP (µmol TE/L)	0.162	-0.304	0.629	4.96E-01	0.244	0.230	-0.231	0.691	3.30E-01	0.303	0.245	-0.197	0.686	2.79E-01	0.363
TEAC-ABTS (µmol TE/L)	0.063	-0.150	0.275	5.62E-01	0.243	0.051	-0.161	0.263	6.40E-01	0.301	0.052	-0.151	0.256	6.16E-01	0.360
Total Polyphenols (mg GAE/L)	-0.569	-1.190	0.051	7.34E-02	0.254	-0.466	-1.113	0.181	1.59E-01	0.307	-0.303	-0.930	0.324	3.45E-01	0.362
ORAC without proteins (µmol TE/L)	0.020	-0.186	0.227	8.48E-01	0.242	0.032	-0.174	0.238	7.64E-01	0.300	0.026	-0.171	0.224	7.95E-01	0.359
ORAC (µmol TE/L)	0.216	-0.061	0.494	1.28E-01	0.251	0.259	-0.015	0.533	6.56E-02	0.312	0.231	-0.033	0.495	8.76E-02	0.368
CRP (mg/L)	-0.011	-0.074	0.052	7.38E-01	0.241	-0.010	-0.078	0.057	7.66E-01	0.300	0.003	-0.063	0.068	9.41E-01	0.358
Adiponectin (µg/ml)	0.048	-0.022	0.119	1.83E-01	0.248	0.036	-0.034	0.107	3.12E-01	0.305	0.016	-0.052	0.084	6.49E-01	0.362
PAI-I (ng/ml)	0.046	-0.070	0.162	4.38E-01	0.244	0.091	-0.027	0.208	1.31E-01	0.308	0.082	-0.031	0.195	1.55E-01	0.365
Resistin (ng/ml)	-0.062	-0.217	0.093	4.34E-01	0.244	-0.060	-0.213	0.092	4.38E-01	0.302	-0.026	-0.174	0.121	7.28E-01	0.359
TNF-α (pg/ml)	-0.005	-0.141	0.131	9.41E-01	0.238	-0.003	-0.137	0.131	9.64E-01	0.330	-0.020	-0.147	0.108	7.64E-01	0.398
IL-8 (pg/ml)	-0.025	-0.111	0.062	5.79E-01	0.224	-0.017	-0.105	0.071	7.03E-01	0.276	-0.006	-0.090	0.079	8.97E-01	0.349
IL-6 (pg/ml)	0.003	-0.077	0.082	9.50E-01	0.242	0.010	-0.069	0.089	8.04E-01	0.300	0.023	-0.053	0.099	5.58E-01	0.360

Plasma biomarkers were log2 transformed.

Model 1: Multiple linear regression adjusted for age (continuous, years), sex (male, females) and centre (Granada, Gipuzkoa).

Model 2: Multiple linear regression adjusted age (continuous, years), sex (male, females) and centre (Granada, Gipuzkoa), BMI (continuous, kg/m^2), physical activity (inactive, moderately inactive and active, active) and smoking status (never, former, current smoker).

Model 3: Multiple linear regression adjusted age (continuous, years), sex (male, females) and centre (Granada, Gipuzkoa), BMI (continuous, kg/m^2), physical activity (inactive, moderately inactive and active, active), smoking status (never, former, current smoker), and total energy intake (continuous, kcal/day).

Positive association are shown in bold.

Table S8: Association between D-NEAC as FRAP without coffee and biomarkers in the EPIC Granada-Gipuzkoa sub-sample (n=210) in multivariate models.

Biomarkers	Model 1					Model 2					Model 3				
	β coeff	CI 95%		p-value	R^2	β coeff	CI 95%		p-value	R^2	β coeff	CI 95%		p-value	R^2
Ascorbic Acid (μmol/L)	0.043	-0.040	0.127	3.11E-01	0.252	0.037	-0.048	0.121	3.95E-01	0.291	0.026	-0.054	0.105	5.26E-01	0.380
Dehydroascorbic Acid (μmol/L)	-0.001	-0.004	0.002	4.05E-01	0.250	-0.001	-0.004	0.002	4.46E-01	0.291	-0.001	-0.004	0.001	3.23E-01	0.382
Total Vitamin C (μmol/L)	0.034	-0.041	0.108	3.76E-01	0.251	0.028	-0.047	0.103	4.66E-01	0.290	0.013	-0.057	0.084	7.10E-01	0.379
Retinol (μmol/L)	-0.074	-0.242	0.094	3.90E-01	0.250	-0.070	-0.241	0.102	4.27E-01	0.291	-0.070	-0.231	0.090	3.92E-01	0.381
α-Tocopherol (μmol/L)	-0.046	-0.161	0.069	4.36E-01	0.250	-0.042	-0.159	0.075	4.84E-01	0.290	-0.025	-0.134	0.085	6.61E-01	0.380
Carotenes (μmol/L)	0.028	-0.029	0.085	3.42E-01	0.251	0.026	-0.033	0.084	3.96E-01	0.291	0.011	-0.044	0.067	6.91E-01	0.379
Q9 (μmol/L)	-0.006	-0.065	0.053	8.42E-01	0.248	-0.004	-0.063	0.055	8.85E-01	0.289	0.011	-0.044	0.067	6.92E-01	0.379
Q10 (μmol/L)	-0.048	-0.155	0.059	3.80E-01	0.251	-0.032	-0.142	0.078	5.69E-01	0.290	-0.025	-0.128	0.078	6.32E-01	0.380
Uric Acid (mg/dl)	-0.098	-0.273	0.077	2.74E-01	0.252	-0.026	-0.212	0.160	7.87E-01	0.289	-0.026	-0.200	0.148	7.69E-01	0.379
FRAP (μmol TE/L)	0.044	-0.273	0.361	7.85E-01	0.248	0.154	-0.171	0.479	3.54E-01	0.292	0.106	-0.199	0.411	4.97E-01	0.380
FRAP (μmol Fe^{2+}/L)	-0.047	-0.384	0.290	7.85E-01	0.248	0.053	-0.291	0.397	7.64E-01	0.289	-0.029	-0.353	0.294	8.59E-01	0.379
FRAP without Uric Acid (μmol TE/L)	0.107	-0.168	0.383	4.47E-01	0.250	0.161	-0.115	0.438	2.54E-01	0.293	0.107	-0.154	0.367	4.23E-01	0.381
FRAP without Uric Acid (μmol Fe^{2+}/L)	0.016	-0.287	0.319	9.19E-01	0.248	0.067	-0.238	0.372	6.68E-01	0.289	-0.028	-0.315	0.260	8.51E-01	0.379
TRAP (μmol TE/L)	0.093	-0.299	0.485	6.43E-01	0.249	0.139	-0.253	0.531	4.88E-01	0.290	0.154	-0.213	0.521	4.11E-01	0.381
TEAC-ABTS (μmol TE/L)	0.066	-0.112	0.244	4.69E-01	0.250	0.058	-0.122	0.238	5.29E-01	0.290	0.060	-0.109	0.228	4.90E-01	0.380
Total Polyphenols (mg GAE/L)	-0.550	-1.069	-0.030	**3.95E-02**	0.263	-0.461	-1.010	0.088	1.01E-01	0.298	-0.290	-0.810	0.230	2.75E-01	0.383
ORAC without proteins (μmol TE/L)	-0.022	-0.195	0.152	8.06E-01	0.248	-0.015	-0.190	0.160	8.68E-01	0.289	-0.021	-0.184	0.144	8.07E-01	0.379
ORAC (μmol TE/L)	0.156	-0.077	0.390	1.91E-01	0.254	0.193	-0.040	0.427	1.06E-01	0.298	0.164	-0.055	0.383	1.44E-01	0.386
CRP (mg/L)	-0.022	-0.074	0.030	4.11E-01	0.252	-0.020	-0.077	0.037	4.95E-01	0.292	-0.007	-0.060	0.047	8.07E-01	0.381
Adiponectin (μg/ml)	0.031	-0.028	0.090	3.03E-01	0.255	0.022	-0.038	0.081	4.78E-01	0.294	-0.000	-0.056	0.056	9.97E-01	0.384
PAI-I (ng/ml)	0.031	-0.067	0.128	5.41E-01	0.249	0.065	-0.035	0.165	2.04E-01	0.294	0.056	-0.038	0.150	2.43E-01	0.383
Resistin (ng/ml)	-0.103	-0.232	0.027	1.22E-01	0.256	-0.100	-0.229	0.029	1.32E-01	0.297	-0.064	-0.186	0.058	3.03E-01	0.382
TNF-α (pg/ml)	0.011	-0.102	0.125	8.46E-01	0.244	0.010	-0.102	0.123	8.57E-01	0.318	-0.007	-0.111	0.098	9.04E-01	0.419
IL-8 (pg/ml)	-0.020	-0.092	0.052	5.86E-01	0.236	-0.016	-0.090	0.058	6.71E-01	0.278	-0.005	-0.074	0.064	8.88E-01	0.371
IL-6 (pg/ml)	-0.010	-0.077	0.057	7.75E-01	0.248	-0.004	-0.071	0.063	9.11E-01	0.289	0.009	-0.054	0.072	7.71E-01	0.379

Plasma biomarkers were log2 transformed.

Model 1: Multiple linear regression adjusted for age (continuous, years), sex (male, females) and centre (Granada, Gipuzkoa).

Model 2: Multiple linear regression adjusted age (continuous, years), sex (male, females) and centre (Granada, Gipuzkoa), BMI (continuous, kg/m^2), physical activity (inactive, moderately inactive and active, active) and smoking status (never, former, current smoker).

Model 3: Multiple linear regression adjusted age (continuous, years), sex (male, females) and centre (Granada, Gipuzkoa), BMI (continuous, kg/m^2), physical activity (inactive, moderately inactive and active, active), smoking status (never, former, current smoker), and total energy intake (continuous, kcal/day).

Positive association are shown in bold.

Table S9: Association between P-NEAC as FRAP and biomarkers in the EPIC Granada-Gipuzkoa sub-sample (n=210) by smoking status.

Biomarkers	Never smoke (n=130)					Ever smoke (n=79)				
	β coeff	CI 95%		p-value	R^2	β coeff	CI 95%		p-value	R^2
Ascorbic Acid (µmol/L)	0.019	-0.009	0.047	1.91E-01	0.323	0.042	0.000	0.083	5.24E-02	0.268
Dehydroascorbic Acid (µmol/L)	-0.000	-0.001	0.001	8.88E-01	0.314	0.001	0.000	0.003	**1.87E-02**	0.287
Total Vitamin C (µmol/L)	0.009	-0.016	0.035	4.77E-01	0.316	0.034	-0.002	0.070	7.16E-02	0.263
Retinol (µmol/L)	0.041	-0.015	0.098	1.56E-01	0.325	0.143	0.062	0.224	**9.12E-04**	0.341
α-Tocopherol (µmol/L)	0.024	-0.016	0.063	2.52E-01	0.321	0.088	0.034	0.143	**2.14E-03**	0.325
Carotenes (µmol/L)	0.009	-0.012	0.030	3.87E-01	0.318	0.030	0.003	0.057	**3.38E-02**	0.276
Q9 (µmol/L)	0.012	-0.007	0.031	2.07E-01	0.322	0.011	-0.025	0.047	5.51E-01	0.231
Q10 (µmol/L)	0.047	0.009	0.084	**1.64E-02**	0.345	0.083	0.034	0.133	**1.49E-03**	0.332
Uric Acid (mg/dl)	0.263	0.217	0.309	**2.50E-20**	0.662	0.239	0.168	0.311	**8.03E-09**	0.521
TRAP (µmol TE/L)	0.045	-0.092	0.182	5.24E-01	0.316	0.269	0.091	0.448	**4.20E-03**	0.313
TEAC-ABTS (µmol TE/L)	0.004	-0.064	0.073	9.07E-01	0.313	-0.041	-0.123	0.041	3.29E-01	0.238
Total Polyphenols (mg GAE/L)	0.314	0.134	0.493	**8.33E-04**	0.374	0.133	-0.136	0.402	3.36E-01	0.238
ORAC without proteins (µmol TE/L)	0.067	0.007	0.126	**2.97E-02**	0.340	-0.048	-0.134	0.037	2.73E-01	0.241
ORAC (µmol TE/L)	0.001	-0.081	0.083	9.78E-01	0.313	-0.115	-0.227	-0.004	**4.60E-02**	0.270
CRP (mg/L)	0.004	-0.017	0.025	7.00E-01	0.316	-0.013	-0.038	0.013	3.33E-01	0.239
Adiponectin (µg/ml)	-0.011	-0.028	0.007	2.38E-01	0.324	0.030	-0.009	0.069	1.40E-01	0.253
PAI-I (ng/ml)	0.012	-0.022	0.046	4.89E-01	0.316	0.006	-0.049	0.060	8.42E-01	0.228
Resistin (ng/ml)	0.001	-0.052	0.053	9.78E-01	0.313	0.008	-0.047	0.062	7.86E-01	0.228
TNF-α (pg/ml)	0.011	-0.031	0.052	6.13E-01	0.313	0.013	-0.039	0.064	6.32E-01	0.185
IL-8 (pg/ml)	-0.012	-0.037	0.012	3.17E-01	0.285	0.000	-0.039	0.039	9.95E-01	0.162
IL-6 (pg/ml)	-0.017	-0.046	0.013	2.64E-01	0.320	-0.014	-0.040	0.013	3.12E-01	0.239

Plasma biomarkers were log2 transformed.

Model 1: Multiple linear regression adjusted for age (continuous, years), sex (male, females) and centre (Granada, Gipuzkoa).

Model 2: Multiple linear regression adjusted age (continuous, years), sex (male, females) and centre (Granada, Gipuzkoa), BMI (continuous, kg/m^2), physical activity (inactive, moderately inactive and active, active).

Missing data: smoking status (n=1).

Positive association are shown in bold.

Table S10: Association between P-NEAC as FRAP and biomarkers in the EPIC Granada-Gipuzkoa sub-sample (n=210) by body mass index (BMI).

**Biomarkers**	**Normal & Overweight (n=152)**					**Obese (n=58)**				
	**β coeff**	**CI 95%**		**p-value**	**R^2**	**β coeff**	**CI 95%**		**p-value**	**R^2**
Ascorbic Acid (µmol/L)	0.029	0.000	0.058	5.19E-02	0.240	0.026	-0.015	0.066	2.17E-01	0.486
Dehydroascorbic Acid (µmol/L)	0.001	0.000	0.001	3.06E-01	0.225	0.002	0.000	0.003	**4.86E-02**	0.511
Total Vitamin C (µmol/L)	0.017	-0.009	0.043	2.04E-01	0.228	0.027	-0.010	0.064	1.59E-01	0.491
Retinol (µmol/L)	0.095	0.038	0.153	**1.47E-03**	0.274	0.065	-0.017	0.147	1.29E-01	0.494
α-Tocopherol (µmol/L)	0.065	0.026	0.104	**1.26E-03**	0.275	0.029	-0.028	0.086	3.27E-01	0.480
Carotenes (µmol/L)	0.021	0.001	0.040	**3.72E-02**	0.243	0.016	-0.020	0.051	3.96E-01	0.477
Q9 (µmol/L)	0.015	-0.005	0.035	1.48E-01	0.231	0.006	-0.026	0.038	7.07E-01	0.471
Q10 (µmol/L)	0.084	0.049	0.119	**4.74E-06**	0.328	0.023	-0.035	0.082	4.38E-01	0.476
Uric Acid (mg/dl)	0.262	0.220	0.304	**1.13E-23**	0.619	0.198	0.117	0.279	**1.60E-05**	0.641
TRAP (µmol TE/L)	0.135	-0.008	0.277	6.56E-02	0.238	0.171	0.003	0.338	5.21E-02	0.510
TEAC-ABTS (µmol TE/L)	0.004	-0.060	0.067	9.04E-01	0.220	-0.036	-0.128	0.057	4.53E-01	0.475
Total Polyphenols (mg GAE/L)	0.272	0.088	0.455	**4.35E-03**	0.263	0.272	-0.011	0.556	6.57E-02	0.506
ORAC without proteins (µmol TE/L)	0.025	-0.036	0.085	4.26E-01	0.223	0.068	-0.016	0.152	1.17E-01	0.496
ORAC (µmol TE/L)	-0.040	-0.119	0.041	3.35E-01	0.225	0.008	-0.121	0.137	9.07E-01	0.469
CRP (mg/L)	-0.001	-0.020	0.018	9.03E-01	0.219	0.011	-0.016	0.037	4.41E-01	0.493
Adiponectin (µg/ml)	-0.009	-0.028	0.010	3.36E-01	0.223	0.013	-0.024	0.051	4.94E-01	0.498
PAI-I (ng/ml)	0.027	-0.010	0.064	1.56E-01	0.231	0.008	-0.034	0.050	7.01E-01	0.471
Resistin (ng/ml)	0.018	-0.025	0.062	4.13E-01	0.223	-0.040	-0.109	0.030	2.69E-01	0.482
TNF-α (pg/ml)	0.026	-0.015	0.067	2.17E-01	0.209	-0.001	-0.052	0.049	9.63E-01	0.389
IL-8 (pg/ml)	-0.008	-0.034	0.018	5.31E-01	0.196	-0.000	-0.033	0.032	9.93E-01	0.399
IL-6 (pg/ml)	-0.021	-0.047	0.005	1.22E-01	0.233	0.007	-0.020	0.035	5.98E-01	0.472

Plasma biomarkers were log2 transformed.

Model 1: Multiple linear regression adjusted for age (continuous, years), sex (male, females) and centre (Granada, Gipuzkoa).

Model 2: Multiple linear regression adjusted age (continuous, years), sex (male, females) and centre (Granada, Gipuzkoa), physical activity (inactive, moderately inactive and active, active), and smoking status (never, former, current smokers).

Missing data: smoking status (n=1).

Positive association are shown in bold.

Table S11: Association between P-NEAC as FRAP and biomarkers in the EPIC Granada-Gipuzkoa sub-sample (n=210) by levels of adherence to the Mediterranean Diet.

Biomarkers	Low and médium adherence (score < 12) (n=140)					High adherence (score > 12) (n=70)				
	β coeff	CI 95%		p-value	R^2	β coeff	CI 95%		p-valor	R^2
Ascorbic Acid (µmol/L)	0.0314	0.003	0.0600	**3.33E-02**	0.2778	0.0086	-0.038	0.0552	7.20E-01	0.4744
Dehydroascorbic Acid (µmol/L)	0.0006	0.000	0.0016	2.22E-01	0.2605	0.0003	-0.001	0.0014	5.42E-01	0.4766
Total Vitamin C (µmol/L)	0.0235	-0.002	0.0492	7.69E-02	0.2699	-0.0026	-0.041	0.0360	8.95E-01	0.4734
Retinol (µmol/L)	0.0878	0.026	0.1494	**5.93E-03**	0.2947	0.0604	-0.015	0.1354	1.20E-01	0.4950
α-Tocopherol (µmol/L)	0.0498	0.010	0.0898	**1.60E-02**	0.2849	0.0479	-0.010	0.1057	1.10E-01	0.4961
Carotenes (µmol/L)	0.0124	-0.010	0.0351	2.89E-01	0.2584	0.0241	0.002	0.0465	**3.95E-02**	0.5107
Q9 (µmol/L)	0.0000	-0.021	0.0211	9.98E-01	0.2519	0.0304	0.006	0.0551	**1.93E-02**	0.5211
Q10 (µmol/L)	0.0642	0.027	0.1016	**9.95E-04**	0.3124	0.0673	0.015	0.1195	**1.42E-02**	0.5255
Uric Acid (mg/dl)	0.2619	0.212	0.3116	**1.34E-18**	0.5906	0.2173	0.155	0.2798	**6.05E-09**	0.7074
TRAP (µmol TE/L)	0.1435	0.007	0.2797	**4.10E-02**	0.2758	0.0451	-0.141	0.2311	6.36E-01	0.4753
TEAC-ABTS (µmol TE/L)	-0.0260	-0.091	0.0387	4.32E-01	0.2555	0.0289	-0.052	0.1098	4.86E-01	0.4777
Total Polyphenols (mg GAE/L)	0.3193	0.127	0.5116	**1.45E-03**	0.3086	0.1287	-0.113	0.3706	3.01E-01	0.4830
ORAC without proteins (µmol TE/L)	0.0466	-0.016	0.1089	1.45E-01	0.2642	0.0237	-0.056	0.1037	5.65E-01	0.4763
ORAC (µmol TE/L)	-0.0251	-0.114	0.0635	5.79E-01	0.2537	-0.0486	-0.149	0.0520	3.48E-01	0.4813
CRP (mg/L)	-0.0028	-0.022	0.017	7.84E-01	0.2529	-0.0051	-0.033	0.0231	7.24E-01	0.4744
Adiponectin (µg/ml)	0.0015	-0.024	0.0275	9.08E-01	0.2510	-0.0040	-0.024	0.0156	6.89E-01	0.4759
PAI-I (ng/ml)	0.0043	-0.032	0.0402	8.13E-01	0.2522	0.0323	-0.013	0.0774	1.66E-01	0.4905
Resistin (ng/ml)	0.0102	-0.034	0.0548	6.54E-01	0.2531	0.0038	-0.061	0.0681	9.09E-01	0.4734
TNF-α (pg/ml)	0.0298	-0.012	0.0720	1.71E-01	0.2361	-0.0120	-0.060	0.0356	6.23E-01	0.4581
IL-8 (pg/ml)	-0.0101	-0.038	0.0183	4.89E-01	0.1817	-0.0153	-0.043	0.0121	2.80E-01	0.5120
IL-6 (pg/ml)	-0.0207	-0.048	0.0061	1.33E-01	0.2649	-0.0171	-0.043	0.0085	1.96E-01	0.4884

Plasma biomarkers were log2 transformed.

Model 1: Multiple linear regression adjusted for age (continuous, years), sex (male, females) and centre (Granada, Gipuzkoa).

Model 2: Multiple linear regression adjusted age (continuous, years), sex (male, females) and centre (Granada, Gipuzkoa), BMI (continuous, kg/m^2), physical activity (inactive, moderately inactive and active, active) and smoking status (never, former, current smoker).

Statistically significant associations are shown in bold.

Table S12: Association between P-NEAC as FRAP and biomarkers in cluster-1, comprising mostly women from EPIC-Granada, who were non-smokers and inactive, n=70. Median FRAP levels = 395.0 µmol TE/L.

Biomarkers	Model 1					Model 2				
	coeff	CILow	CIHigh	p-value	R-squared	coeff	CILow	CIHigh	p-value	R-squared
Ascorbic Acid (µmol/L)	0,021	-0,018	0,061	2,93E-01	0,104	0,011	-0,031	0,053	6,18E-01	0,164
Dehydroascorbic Acid (µmol/L)	0,000	-0,002	0,002	8,98E-01	0,088	-0,000	-0,002	0,002	7,66E-01	0,162
Total Vitamin C (µmol/L)	0,023	-0,016	0,060	2,58E-01	0,106	0,012	-0,030	0,054	5,77E-01	0,165
Retinol (µmol/L)	0,037	-0,044	0,118	3,73E-01	0,099	0,045	-0,043	0,132	3,18E-01	0,174
α-Tocopherol (µmol/L)	0,013	-0,054	0,080	7,07E-01	0,090	0,017	-0,056	0,090	6,52E-01	0,163
Carotenes (µmol/L)	-0,011	-0,039	0,018	4,59E-01	0,096	-0,010	-0,040	0,020	5,25E-01	0,166
Q9 (µmol/L)	0,007	-0,017	0,031	5,60E-01	0,093	0,009	-0,016	0,034	4,89E-01	0,167
Q10 (µmol/L)	0,002	-0,048	0,053	9,29E-01	0,088	0,005	-0,051	0,061	8,72E-01	0,161
Uric Acid (mg/dl)	0,206	0,152	0,260	**2,62E-10**	0,509	0,222	0,164	0,281	**4,33E-10**	0,568
TRAP (µmol TE/L)	-0,062	-0,266	0,142	5,54E-01	0,093	-0,047	-0,260	0,166	6,68E-01	0,163
TEAC-ABTS (µmol TE/L)	-0,059	-0,197	0,079	4,03E-01	0,098	-0,033	-0,180	0,113	6,56E-01	0,163
Total Polyphenols (mg GAE/L)	0,202	-0,043	0,446	1,11E-01	0,123	0,255	-0,004	0,513	5,85E-02	0,210
ORAC without proteins (µmol TE/L)	0,107	0,019	0,196	**2,05E-02**	0,161	0,091	-0,010	0,191	8,29E-02	0,202
ORAC (µmol TE/L)	-0,099	-0,200	0,002	6,01E-02	0,137	-0,095	-0,205	0,015	9,63E-02	0,199
CRP (mg/L)	0,020	-0,002	0,040	8,78E-02	0,148	0,030	0,007	0,053	**1,45E-02**	0,296
Adiponectin (pg/ml)	-0,022	-0,037	-0,007	**5,45E-03**	0,211	-0,020	-0,035	-0,005	**1,20E-02**	0,293
PAI-I (pg/ml)	0,033	-0,013	0,080	1,66E-01	0,115	0,037	-0,013	0,087	1,57E-01	0,189
Resistin (pg/ml)	0,072	0,011	0,132	**2,38E-02**	0,157	0,078	0,012	0,145	**2,51E-02**	0,229
TNF-α (pg/ml)	0,032	-0,006	0,069	1,05E-01	0,171	0,015	-0,024	0,054	4,52E-01	0,325
IL-8 (pg/ml)	0,010	-0,013	0,034	4,02E-01	0,106	0,007	-0,018	0,032	5,90E-01	0,199
IL-6 (pg/ml)	-0,012	-0,037	0,013	3,52E-01	0,100	-0,012	-0,041	0,017	4,14E-01	0,170

Plasma biomarkers were log2 transformed.

Model 1: Multiple linear regression adjusted for age (continuous, years), sex (male, females) and centre (Granada, Gipuzkoa).

Model 2: Multiple linear regression adjusted age (continuous, years), sex (male, females) and centre (Granada, Gipuzkoa), BMI (continuous, kg/m^2), physical activity (inactive, moderately inactive and active, active) and smoking status (never, former, current smoker).

Statistically significant associations are shown in bold.

Table S13: Sensitivity analyses comprising the removal of influential points based on Cook´s distances in multivariate models.

**Biomarkers**	**TRAP**					**FRAP**				
	**β coeff**	**CI 95%**		**p-value**	**R^2**	**β coeff**	**CI 95%**		**p-value**	**R^2**
Ascorbic Acid (µmol/L)	-0.025	-0.046	-0.004	**2.02E-02**	0.232	0.027	0.006	0.048	**1.33E-02**	0.335
Dehydroascorbic Acid (µmol/L)	0.000	-0.001	0.001	9.57E-01	0.204	0.001	0.000	0.001	1.61E-01	0.336
Total Vitamin C (µmol/L)	-0.016	-0.035	0.003	9.28E-02	0.221	0.018	-0.001	0.036	7.07E-02	0.325
Retinol (µmol/L)	-0.012	-0.056	0.031	5.80E-01	0.220	0.059	0.017	0.102	**6.99E-03**	0.361
α-Tocopherol (µmol/L)	-0.017	-0.048	0.013	2.61E-01	0.221	0.045	0.016	0.075	**3.15E-03**	0.367
Carotenes (µmol/L)	-0.004	-0.019	0.011	6.10E-01	0.217	0.022	0.007	0.036	**3.56E-03**	0.351
Q9 (µmol/L)	-0.011	-0.025	0.004	1.52E-01	0.220	0.015	0.000	0.029	5.19E-02	0.333
Q10 (µmol/L)	-0.006	-0.035	0.024	7.03E-01	0.216	0.058	0.029	0.087	**1.02E-04**	0.372
Uric Acid (mg/dl)	0.074	0.026	0.122	**2.68E-03**	0.246	0.230	0.195	0.265	**3.93E-27**	0.636
FRAP (µmol TE/L)	0.187	0.104	0.270	**1.82E-05**	0.305	-	-	-	**-**	-
FRAP (µmol Fe^{2+}/L)	0.127	0.029	0.224	**1.16E-02**	0.232	-	-	-	**-**	-
FRAP without Uric Acid (µmol TE/L)	0.149	0.079	0.219	**4.84E-05**	0.272	-	-	-	**-**	-
FRAP without Uric Acid (µmol Fe^{2+}/L)	0.077	-0.010	0.163	8.29E-02	0.218	-	-	-	**-**	-
TRAP (µmol TE/L)	-	-	-	**-**	-	0.127	0.029	0.224	**1.16E-02**	0.363
TEAC-ABTS (µmol TE/L)	0.074	0.030	0.118	**1.10E-03**	0.243	-0.013	-0.059	0.033	5.85E-01	0.331
Total Polyphenols (mg GAE/L)	0.160	0.022	0.297	**2.44E-02**	0.233	0.198	0.060	0.337	**5.52E-03**	0.350
ORAC without proteins (µmol TE/L)	0.039	-0.004	0.081	7.46E-02	0.226	0.031	-0.012	0.074	1.56E-01	0.335
ORAC (µmol TE/L)	0.068	0.010	0.126	**2.37E-02**	0.240	-0.040	-0.098	0.017	1.72E-01	0.354
CRP (mg/L)	0.001	-0.014	0.016	9.16E-01	0.205	-0.004	-0.019	0.012	6.46E-01	0.326
Adiponectin (µg/ml)	0.006	-0.012	0.025	4.96E-01	0.220	-0.001	-0.019	0.018	9.33E-01	0.331
PAI-I (ng/ml)	0.014	-0.013	0.042	3.06E-01	0.207	-0.010	-0.037	0.018	4.99E-01	0.328
Resistin (ng/ml)	-0.031	-0.064	0.001	6.26E-02	0.212	-0.004	-0.036	0.029	8.32E-01	0.354
TNF-α (pg/ml)	-0.025	-0.056	0.008	1.36E-01	0.261	0.040	0.009	0.070	**1.27E-02**	0.382
IL-8 (pg/ml)	-0.007	-0.025	0.011	4.31E-01	0.308	-0.003	-0.021	0.015	7.54E-01	0.337
IL-6 (pg/ml)	-0.003	-0.020	0.015	7.73E-01	0.210	-0.019	-0.037	-0.002	**2.98E-02**	0.347

Association between P-NEAC as TRAP and as FRAP and biomarkers in the EPIC Granada-Gipuzkoa cohort sub-sample (n=210).

Plasma biomarkers were log2 transformed.

Multiple linear regression adjusted age (continuous, years), sex (male, females) and centre (Granada, Gipuzkoa), BMI (continuous, kg/m^2), physical activity (inactive, moderately inactive and active, active) and smoking status (never, former, current smoker).

Statistically significant associations are shown in bold.

Table S14: Association between P-NEAC as FRAP without uric acid and biomarkers in the EPIC Granada-Gipuzkoa cohort sub-sample (n=210).

Biomarkers	Model 1					Model 2				
	**β coeff**	**CI 95%**		**p-value**	**R^2**	**β coeff**	**CI 95%**		**p-value**	**R^2**
Ascorbic Acid (µmol/L)	0.044	0.018	0.070	**8.97E-04**	0.212	0.043	0.017	0.069	**1.37E-03**	0.237
Dehydroascorbic Acid (µmol/L)	0.001	0.000	0.001	1.76E-01	0.175	0.001	0.000	0.001	1.58E-01	0.205
Total Vitamin C (µmol/L)	0.031	0.008	0.054	**7.89E-03**	0.196	0.031	0.007	0.054	**1.09E-02**	0.223
Retinol (µmol/L)	0.076	0.024	0.128	**4.49E-03**	0.200	0.080	0.027	0.133	**3.66E-03**	0.230
α-Tocopherol (µmol/L)	0.050	0.014	0.085	**6.46E-03**	0.198	0.050	0.014	0.087	**7.30E-03**	0.226
Carotenes (µmol/L)	0.021	0.004	0.039	**1.90E-02**	0.190	0.021	0.003	0.040	**2.35E-02**	0.217
Q9 (µmol/L)	0.016	-0.002	0.034	8.62E-02	0.180	0.014	-0.004	0.033	1.34E-01	0.206
Q10 (µmol/L)	0.061	0.028	0.093	**3.18E-04**	0.219	0.064	0.030	0.097	**2.78E-04**	0.249
Uric Acid (mg/dl)	0.152	0.102	0.203	**1.71E-08**	0.288	0.162	0.108	0.216	**2.12E-08**	0.315
TRAP (µmol TE/L)	0.139	0.018	0.260	**2.59E-02**	0.188	0.120	-0.004	0.243	5.85E-02	0.211
TEAC-ABTS (µmol TE/L)	-0.057	-0.113	-0.002	**4.34E-02**	0.184	-0.056	-0.113	0.000	5.25E-02	0.212
Total Polyphenols (mg GAE/L)	0.250	0.089	0.411	**2.67E-03**	0.204	0.244	0.072	0.415	**5.88E-03**	0.227
ORAC without proteins (µmol TE/L)	0.010	-0.044	0.065	7.08E-01	0.169	0.011	-0.044	0.067	6.93E-01	0.197
ORAC (µmol TE/L)	-0.056	-0.129	0.017	1.32E-01	0.177	-0.059	-0.133	0.015	1.21E-01	0.206
CRP (mg/L)	-0.005	-0.021	0.011	5.52E-01	0.165	-0.008	-0.025	0.010	4.09E-01	0.196
Adiponectin (µg/ml)	-0.004	-0.022	0.014	6.63E-01	0.164	-0.003	-0.021	0.016	7.81E-01	0.193
PAI-I (ng/ml)	0.002	-0.028	0.033	8.77E-01	0.168	-0.001	-0.032	0.031	9.69E-01	0.197
Resistin (ng/ml)	0.017	-0.023	0.058	4.06E-01	0.171	0.015	-0.026	0.056	4.66E-01	0.199
TNF-α (pg/ml)	0.022	-0.012	0.057	2.07E-01	0.152	0.020	-0.015	0.056	2.68E-01	0.186
IL-8 (pg/ml)	-0.007	-0.028	0.014	5.00E-01	0.166	-0.011	-0.033	0.011	3.35E-01	0.183
IL-6 (pg/ml)	-0.016	-0.037	0.005	1.32E-01	0.177	-0.017	-0.038	0.004	1.14E-01	0.207

Plasma biomarkers were log2 transformed.

Model 1: Multiple linear regression adjusted for age (continuous, years), sex (male, females) and centre (Granada, Gipuzkoa).

Model 2: Multiple linear regression adjusted age (continuous, years), sex (male, females) and centre (Granada, Gipuzkoa), BMI (continuous, kg/m^2), physical activity (inactive, moderately inactive and active, active) and smoking status (never, former, current smoker).

Statistically significant associations are shown in bold.
